# The Complications of Myopia: A Review and Meta-Analysis

**DOI:** 10.1167/iovs.61.4.49

**Published:** 2020-04-29

**Authors:** Annechien E. G. Haarman, Clair A. Enthoven, J. Willem L. Tideman, Milly S. Tedja, Virginie J. M. Verhoeven, Caroline C. W. Klaver

**Affiliations:** 1 Department of Ophthalmology, Erasmus University Medical Centre, Rotterdam, The Netherlands; 2 Department of Epidemiology, Erasmus University Medical Centre, Rotterdam, The Netherlands; 3 Department of Clinical Genetics, Erasmus University Medical Centre, Rotterdam, The Netherlands; 4 Department of Ophthalmology, Radboud University Medical Centre, Nijmegen, Gelderland, The Netherlands; 5 Institute of Molecular and Clinical Ophthalmology, Basel, Switzerland

**Keywords:** myopia, myopic macular degeneration, retinal detachment, cataract, open angle glaucoma

## Abstract

**Purpose:**

To determine the risk between degree of myopia and myopic macular degeneration (MMD), retinal detachment (RD), cataract, open angle glaucoma (OAG), and blindness.

**Methods:**

A systematic review and meta-analyses of studies published before June 2019 on myopia complications. Odds ratios (OR) per complication and spherical equivalent (SER) degree (low myopia SER < –0.5 to > –3.00 diopter [D]; moderate myopia SER ≤ –3.00 to > –6.00 D; high myopia SER ≤ –6.00 D) were calculated using fixed and random effects models.

**Results:**

Low, moderate, and high myopia were all associated with increased risks of MMD (OR, 13.57, 95% confidence interval [CI], 6.18–29.79; OR, 72.74, 95% CI, 33.18–159.48; OR, 845.08, 95% CI, 230.05–3104.34, respectively); RD (OR, 3.15, 95% CI, 1.92–5.17; OR, 8.74, 95% CI, 7.28–10.50; OR, 12.62, 95% CI, 6.65–23.94, respectively); posterior subcapsular cataract (OR, 1.56, 95% CI, 1.32–1.84; OR, 2.55, 95% CI, 1.98–3.28; OR, 4.55, 95% CI, 2.66–7.75, respectively); nuclear cataract (OR, 1.79, 95% CI, 1.08–2.97; OR, 2.39, 95% CI, 1.03–5.55; OR, 2.87, 95% CI, 1.43–5.73, respectively); and OAG (OR, 1.59, 95% CI, 1.33–1.91; OR, 2.92, 95% CI, 1.89–4.52 for low and moderate/high myopia, respectively). The risk of visual impairment was strongly related to longer axial length, higher myopia degree, and age older than 60 years (OR, 1.71, 95% CI, 1.07–2.74; OR, 5.54, 95% CI, 3.12–9.85; and OR, 87.63, 95% CI, 34.50–222.58 for low, moderate, and high myopia in participants aged >60 years, respectively).

**Conclusions:**

Although high myopia carries the highest risk of complications and visual impairment, low and moderate myopia also have considerable risks. These estimates should alert policy makers and health care professionals to make myopia a priority for prevention and treatment.

Myopia or nearsightedness is a refractive error caused by excessive axial elongation.^1^^,^^2^ Myopia can be corrected optically by glasses, contact lenses, or refractive surgery. Nevertheless, it has been associated with complications, such as myopic macular degeneration (MMD), retinal detachment (RD), cataract, and open angle glaucoma (OAG).^3^ These complications can lead to irreversible visual impairment later in life.^4^

The most important complication of myopia is MMD, which is a common cause of visual impairment, particularly for high myopia.^5^ Characteristics of MMD are lacquer cracks, Fuchs spot, choroidal neovascularization (CNV), or chorioretinal atrophy.^6^ Posterior staphyloma is sometimes considered a specific type of MMD, whereas others consider it rather a risk factor for developing MMD.^6^^,^^7^ Common peripheral retinal lesions in high myopia patients are RD, pigmentary degeneration, lattice degeneration, and pavingstone degeneration, of which RD is the most sight-threatening.^5^^,^^8^ For cataract, the relationship with myopia is less evident. In particular, nuclear cataract may result in a myopic shift, which hampers determination of the original refractive error.^9^ Considering OAG, Perkins et al.^10^ already published in 1982 about a higher percentage of myopic patients in the OAG population. A meta-analysis performed on 11 population-based studies also identified an increased risk of OAG for myopic persons.^11^ Whether visual field progression in myopes is similar to other OAG patients is still unclear.

High myopia (spherical equivalent [SER] ≤ –6 D) is associated with reduced vision-related quality of life and has significant socioeconomic impact.^12^ The incidence of myopia and high myopia is rising globally, and it is expected that the burden of its complications will lead to considerable visual morbidity in the near future.^13^^,^^14^ Myopia is already the most common cause of irreversible visual impairment in the working population. A recent study estimated $6 billion global productivity loss due to MMD, and this financial burden will undoubtedly become worse in the coming decades.^15^^,^^16^

Although the association with myopic complications has been well established, precise risk estimates of MMD, RD, cataract, and OAG per degree of myopia are yet unknown.^17^ In this review, we aim to provide a systematic review of the visual morbidity of myopia. First, we calculated the risk estimates of the most prevalent complications, that is, MMD, RD, cataract, and OAG, by performing meta-analyses on all existing data. Because data on other myopia-related complications, such as posterior staphyloma, retinoschisis, and dome-shaped macula, are limited, we did not include these in our review. Second, we explored the impact of these complications on best-corrected visual acuity (BCVA). Considering that cataract can be surgically treated, we also investigated whether this procedure is safe and effective in myopic patients. The risk estimates derived from this study may create awareness among eye care providers for vision-threatening complications associated with myopia and help to inform myopic patients.

## Methods

We followed the guidelines of the PRISMA (Preferred Reporting Items for Systematic Reviews and Meta-Analyses) statement for the meta-analyses.^18^ As published literature was used, ethical approval was not required.

### Search Methods

We conducted an extensive literature search in PubMed on myopia and myopia-related complications using the following MeSH terms: “myopia,” “myopia, degenerative,” “visual acuity,” “retinal degeneration,” “choroidal neovascularization,” “retinal detachment,“ “cataract,” and “glaucoma.” The complete PubMed search strategy is available in Supplementary Table S1, and the PRISMA flow diagram is available in Supplementary Figure S1. Titles and abstracts of articles, published before June 1, 2019, were independently reviewed for relevancy by two authors (AEGH and CAE) and included when the following criteria were met: (1) full text available; (2) written in English; and (3) subject of article was myopia complications, visual consequences of myopia, epidemiology of myopia, or epidemiology of visual impairment. Any discrepancies between the two authors were solved by a thorough discussion with other experts until consensus was reached. A manual search was additionally performed by screening of the references of the included articles. All observational studies were considered for inclusion in the meta-analyses.

### Data Extraction and Quality Assessment

We obtained (1) geographic region of data collection; (2) period of data collection; (3) risk estimates of MMD, RD, cataract, and OAG for myopia and different myopia categories; and (4) visual acuity (VA) data of myopic patients with and without complications from each selected study. We assessed the quality of all studies using the criteria proposed by Sanderson et al.^19^ The variables examined included the definitions of the exposures (any, low, moderate, and high myopia), definitions of the outcome variables (MMD, RD, cataract, and OAG), number of participants, age ranges, sex prevalence, study design, and confounding factors used for adjustment. Crude odds ratios (ORs) were calculated for MMD when they were not reported in the studies, using the following formula:

$$\begin{array}{c} OR=\frac{\mathrm{myope}\;\mathrm{with}\;\mathrm{complication}/\mathrm{myope}\;\mathrm{without}\;\mathrm{complication}}{\mathrm{emmetrope}\;\mathrm{with}\;\mathrm{complication}/\mathrm{emmetrope}\;\mathrm{without}\;\mathrm{complication}} \end{array}$$

If the number of cases was zero, it was set to 1 for the OR calculation. Refractive error was categorized into five groups: no myopia (SER > –0.5 diopter D]), any myopia (SER ≤ –0.5 D), low myopia (SER < –0.5 to > –3.00 D), moderate myopia (SER ≤ –3.00 to > –6.00 D), and high myopia (SER ≤ –6.00 D), in line with the most recent classification system.^20^

### Data Syntheses

Meta-analyses were performed using a previously validated method in Microsoft Excel 2010 (Microsoft, Redmond, WA, USA); forest plots for all complications and myopia categories were constructed in GraphPad Prism 5 (GraphPad, San Diego, CA, USA).^21^ A fixed or random effects model was used depending on the number of included studies and the critical value of the calculated Q statistic on the χ^2^ distribution. The Q statistic was calculated as the weighted sum of squared differences between individual study effects and the pooled effect across different studies. We calculated I^2^ to investigate heterogeneity between studies, using the formula: ((Q-*df*)/Q)*100% (*df* represents degrees of freedom). We used a fixed effects model if heterogeneity was low, that is, the calculated Q was lower than the critical value on a χ^2^ distribution, and we used a random effects model otherwise.^21^ Heterogeneity was considered as no, low, moderate, or high for values of <25%, 25% to 50%, 50% to 75%, and ≥75%, respectively.^22^

## Results

### Myopic Macular Degeneration

#### Prevalence of MMD

**Figure 1.**
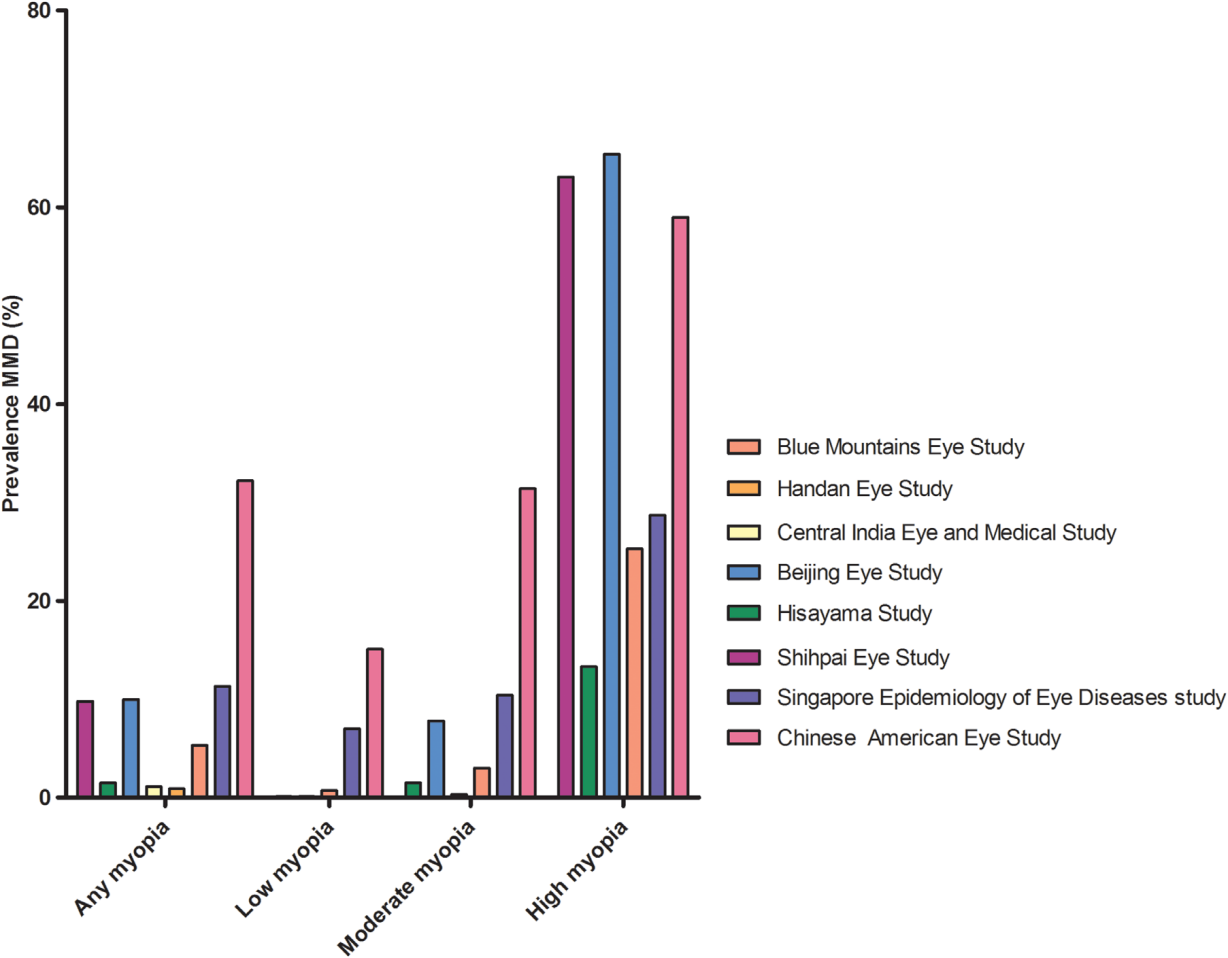
Prevalence of MMD among groups with any, low, moderate, and high myopia derived from eight population-based studies.

The prevalence of MMD in population-based studies varied from 0.2% in rural central India, to 1.2% in Caucasian Australians, and 4.0% in the Singapore Epidemiology of Eye Diseases (SEED) study (Table 1).^23^^–^^30^ Definitions of MMD differed slightly among studies (Supplementary Table S2). After stratification for myopia degree, the prevalence ranged from 13.3% to 65.4% in high myopes, 0.3% to 7.8% in moderate myopes, and 0.1% to 7.0% in low myopes (Fig. 1).^23^^–^^30^ In six nonpopulation-based studies focusing on high myopia patients only, MMD prevalence ranging from 8.3% to 64.0% was reported (Supplementary Table S3).^31^^–^^36^ A remarkably low MMD prevalence (<15%) among high myopia patients was reported in two studies.^33^^,^^37^ The first study was performed in a very young population, Singaporean men aged 19 to 25 years, and the second study was performed in asymptomatic Chinese patients aged 18 years and older, possibly explaining the low prevalence.^33^^,^^37^ The study of Zhao et al.^36^ included the most myopic and oldest participants of which 96.9% had at least a tessellated fundus, and 54.5% also had diffuse, patchy, or macular atrophy.

**Table 1.** Characteristics of the Studies Investigating the Relationship Between Myopia and MMD

Study	Authors	Country	Region	Data Collection Period	Total participants (*n*)	Study type	Age, y^*^	Male Sex (%)	Definition of Myopia (D)	Myopia (%)	High myopia (%)	Total MMD (%)	MMD Definition (Supplementary Table S2)
Blue Mountains Eye Study	Vongphanit et al.^23^ (2002)	Australia	Urban	1992–1993	3583	Prospective	67 (49–97)	43.5	Low: –1 to –3 Moderate: –3 to –5 High: ≤ –5	16.8	2.7	1.2	a (excluding tessellation)
Beijing Eye Study	Liu et al.^24^ (2010)	China	53.9% urban, 46.1% rural	2001	4319	Prospective	57 (40–101)	45.8	Low: –0.5 to –2 Moderate: –2 to –6 High: ≤ –6	23.3	2.4	3.1	a (excluding tessellation)
Handan Eye Study	Gao et al.^25^ (2011)	China	Rural	2006–2007	6603	Prospective	52 (>29)	46.4	Moderate: –0.5 to –5 High: ≤ –5	26.6	2.1	0.9	a (excluding tessellation)
Shihpai Eye Study	Chen et al.^28^ (2012)	Taiwan	Urban	1999–2000	1058	Prospective	72 (65–91)	60.4	Any: ≤ –0.5 High: ≤ –6	30.8	4.2	3.0	b (≥M3; excluding tessellation)
Central India Eye and Medical Study	Jonas et al.^27^ (2017)	India	Rural	2006–2009	4561	Prospective	49 (30–100)	46.3	Any: ≤ –1 High: ≤ –8	16.6	0.5	0.02	c (excluding tessellation)
Hisayama Study	Asakuma et al.^26^ (2012)	Japan	Urban	2005	1892	Prospective	64 (>39)	41.0	Low: 0 to –2 Moderate: –2 to –6 High: ≤ –6	49.0	3.7	1.7	d (excluding tessellation)
Chinese American Eye Study	Choudhury et al.^30^ (2018)	United States	Urban	2010–2013	4582	Prospective	– (<49)	63	Low: –0.5 to –2 Moderate: –2 to –5 High: ≤ –5	32.2	8.0	3.1	c (excluding tessellation)
Singapore Epidemiology of Eye Diseases (SEED) Study	Wong et al.^29^ (2018)	Singapore	Urban	2004–2011	8716	Prospective	57 (40–80)	49.6	Low: –0.5 to –3 Moderate: –3 to –5 High: ≤ –5	35.7	6.0	4.0	c (excluding tessellation)

* Mean (range).

**Figure 2.**
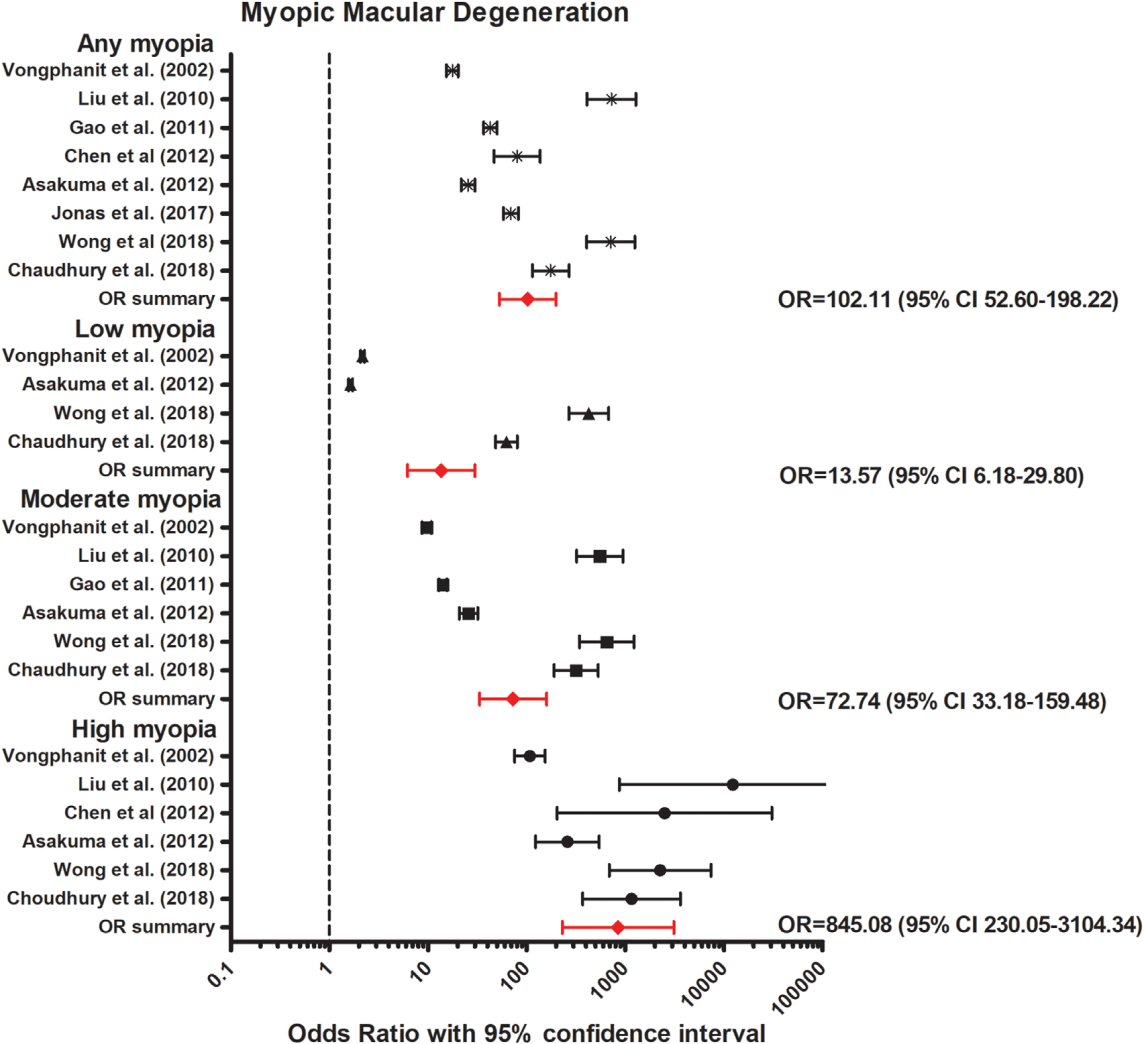
Forest plot of MMD in any myopia (random effects model; Q = 16.1; I^2^ = 56.5); low myopia (random effects model; Q = 27.6; I^2^ = 85.5); moderate myopia (random effects model; Q = 18.0; I^2^ = 72.2), and high myopia (random effects model; Q = 5.2; I^2^ = 4.3). *Red lines with diamond* represents the summary OR per myopia category. Summary OR for myopia categories are as follows: any myopia OR, 102.11 (95% CI, 52.60–198.22); low myopia OR, 13.57 (95% CI, 6.18–29.79); moderate myopia OR, 72.74 (95% CI, 33.18–159.48); and high myopia OR, 845.08 (95% CI, 230.05–3104.34).

Our meta-analyses, including eight population-based studies, revealed an increased OR for any myopia (OR, 102.11; 95% confidence interval [CI], 52.60–198.22, moderate heterogeneity); low myopia (OR, 13.57; 95% CI, 6.18–29.79, high heterogeneity); moderate myopia (OR, 72.74; 95% CI, 33.18–159.48, moderate heterogeneity); and high myopia (OR, 845.08; 95% CI, 230.05–3104.34, no heterogeneity) (Fig. 2).^23^^–^^30^ The association between axial length (AL) and MMD was investigated in three studies. In a Russian population-based study, patients with MMD had a 1.22 mm increased AL compared with those without MMD.^38^ In the Chinese American Eye Study, 80.4% of the participants in the fourth quartile of AL (AL ≥25.60 mm) had a particular lesion (MMD including tessellation, tilted disc, and parapapillary atrophy), whereas in the third (AL 24.65–25.60 mm), second (AL 23.85–24.65 mm), and first quartile (AL <23.85 mm) the percentage was 50.1%, 31.9%, and 17.3%, respectively.^30^ In the Hisayama study, MMD (excluding tessellation, tilted disc, and parapapillary atrophy) was only observed in eyes ≥23.0 mm in men and ≥22.0 mm in women, and the discriminating ability for the presence of MMD was highest at 25.9 mm in men and 25.3 mm in women.^39^

#### Visual Burden of MMD

**Figure 3.**
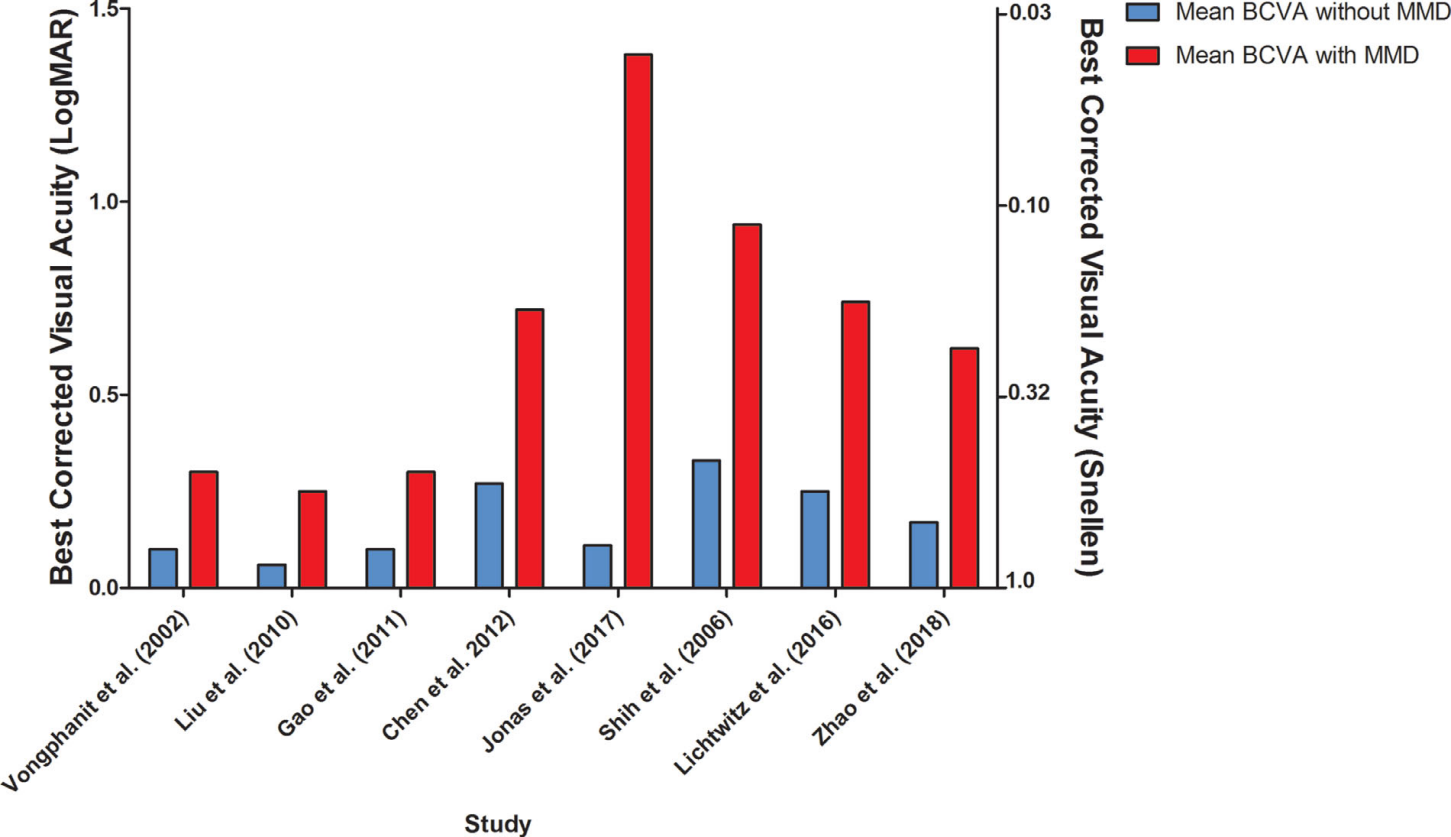
BCVA in eyes with and without MMD.

**Figure 4.**
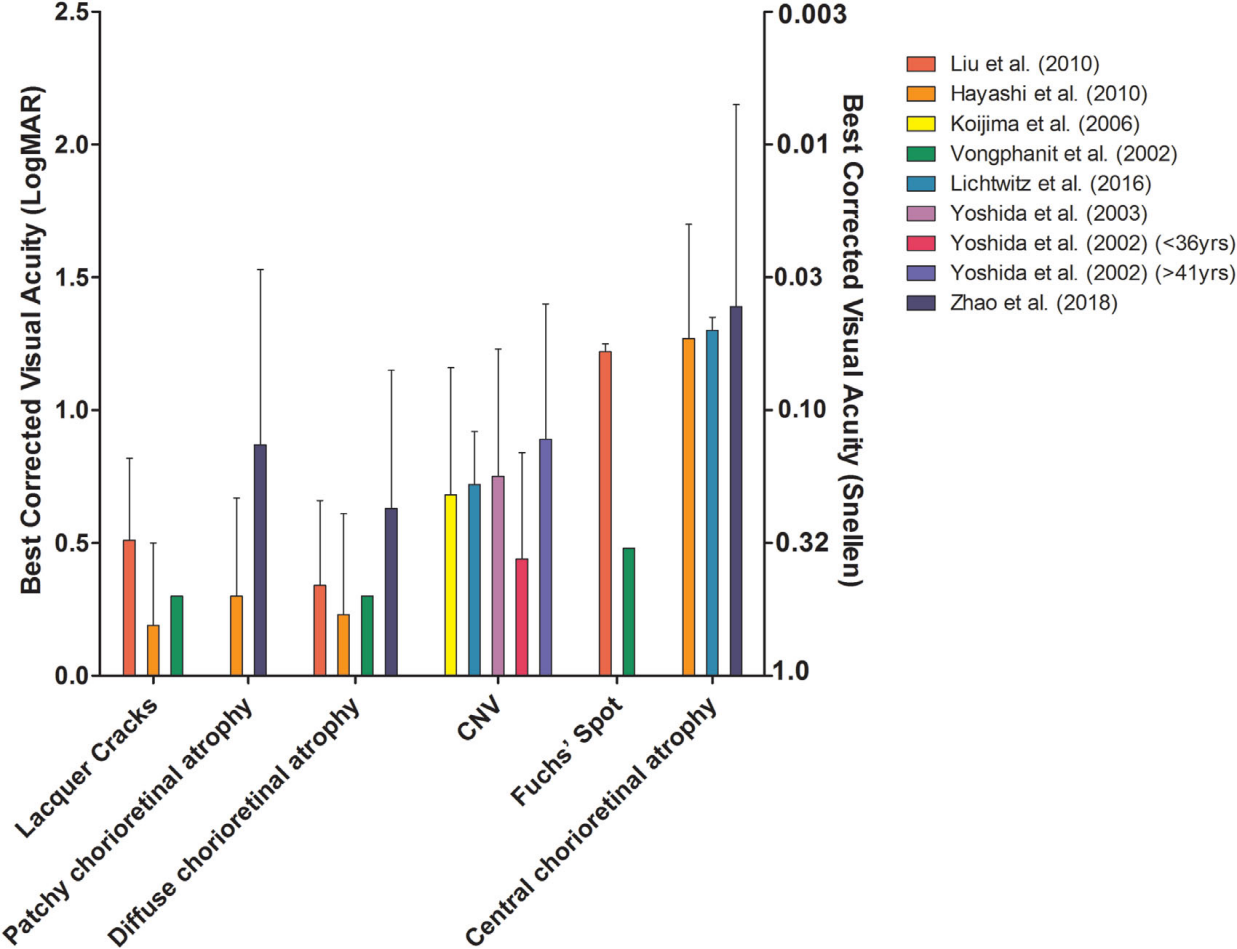
BCVA in eyes with different stages of MMD.

BCVA was measured in eight studies; they all showed a worse BCVA in eyes with MMD compared with eyes without MMD (Supplementary Table S4; Fig. 3).^23^^–^^25^^,^^27^^,^^28^^,^^36^^,^^40^^,^^41^ Macular atrophy had the largest impact on BCVA, followed by CNV, patchy atrophy, diffuse atrophy, or lacquer cracks according to a longitudinal study of MMD patients in Japan. Patients with only a tessellated fundus did not have a decreased BCVA.^42^ Other studies also reported that patients with macular atrophy, CNV, or Fuchs spot had worse BCVA compared with those with patchy or diffuse atrophy, lacquer cracks, or tessellated fundus (Fig. 4).^23^^–^^25^^,^^36^^,^^41^^,^^43^ Progression of MMD to more severe stages was more frequent in older patients.^42^

### Retinal Detachment

#### Incidence of RD

**Figure 5.**
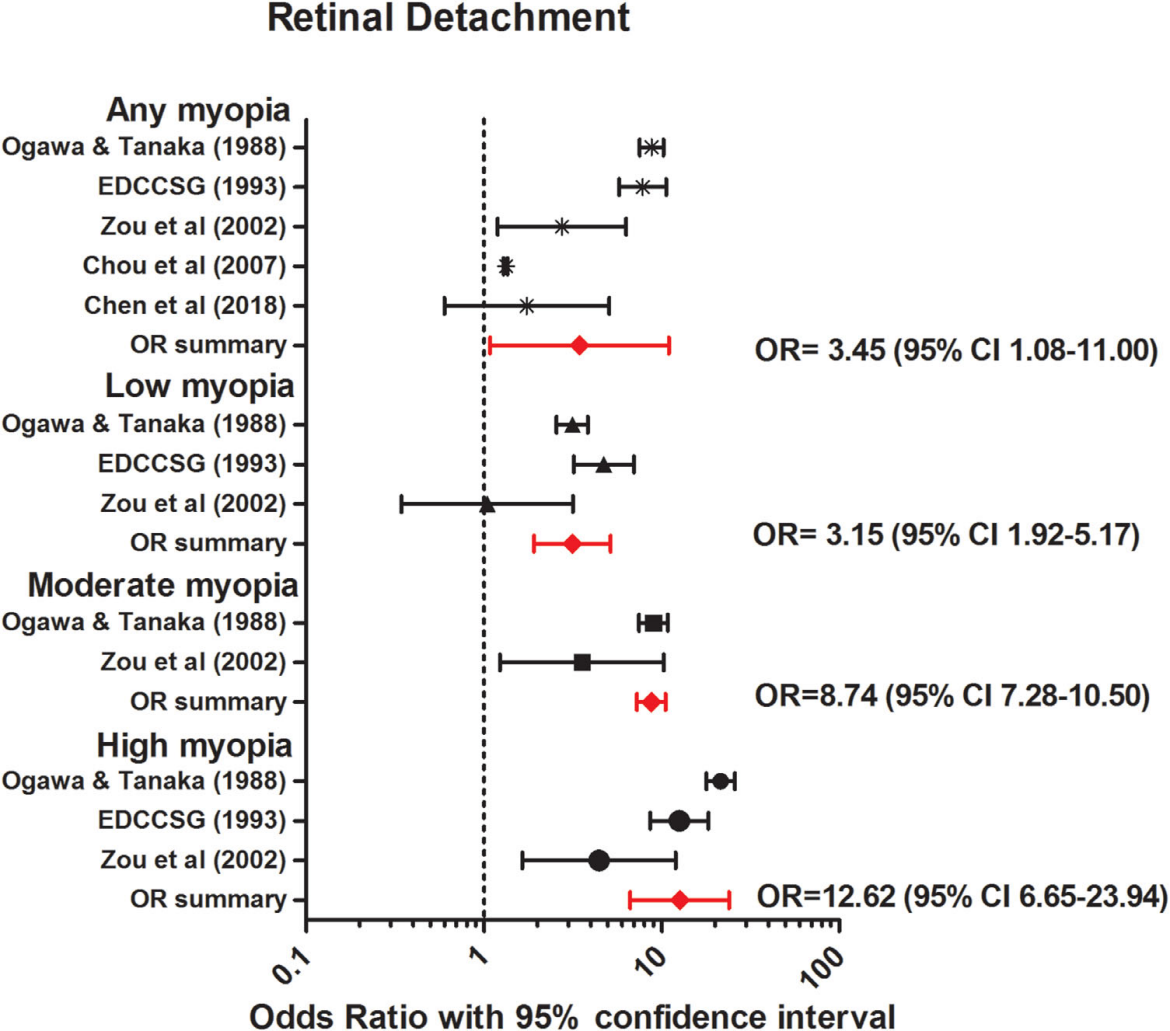
Forest plot of RD in any myopia (random effects model; Q = 1.7; I^2^ = 0.0); low myopia (random effects model; Q = 3.7; I^2^ = 0.5); moderate myopia (fixed effects model; Q = 2.8; I^2^ = 0.6); and high myopia (random effects model; Q = 3.3; I^2^ = 0.4). *Red lines with diamond* represents the summary OR per myopia category. Summary OR for myopia categories are as follows: any myopia OR, 3.45 (95% CI, 1.08–11.00); low myopia OR, 3.15 (95% CI, 1.92–5.17); moderate myopia OR, 8.74 (95% CI, 7.28–10.50); and high myopia OR, 12.62 (95% CI, 6.65–23.94).

Annual incidence rates of RD ranged from 5.4 per 100,000 persons in Croatia (95% CI, 4.1–6.4), to 16.5 per 100,000 persons in Japan (95% CI, 15.0–18.1) (Table 2).^44^^,^^47^^,^^117^^,^^118^^,^^120^^–^^126^ Annual incidence of RD per degree of refractive error was only investigated by Burton et al.,^44^ reporting increased incidence rates of RD with decreasing SER from 3 in 100,000 persons with hyperopia (>0 D), to 102 in 100,000 persons with high myopia (< –9 D) (Table 2). Five case-control studies were available for meta-analyses to determine the relationship between myopia and RD in various refractive error categories (Table 3).^45^^–^^49^ All but one study showed a significant higher odds of RD for myopic persons (<0 D) compared with nonmyopic persons (Fig. 5).^45^^–^^49^ Pooled analyses revealed an increased OR for any myopia (OR, 3.45; 95% CI, 1.08–11.00, no heterogeneity); low myopia (OR, 3.15; 95% CI, 1.92–5.17, no heterogeneity); moderate myopia (OR, 8.74, 95% CI, 7.28–10.50, no heterogeneity); and high myopia (OR, 12.62; 95% CI, 6.65–23.94, no heterogeneity).

**Table 2.** Annual Incidence of RD

Authors	Country	Data Collection Period	Total RD Cases	Male Sex (%)	Age Cases, y^*^	Annual Incidence per 100,000
Laatikainen et al.^121^ (1985)	Finland	1978–1981	310	48.7	54.2 ± 1.0 (5.7–83.0)	6.9 (5.5–8.7)
Törnquist et al.^126^ (1987)	Sweden	1971–1975	590	46.6	59.5 (–)	9.8
		1976–1980				11.4
Li et al.^122^ (2003)	China	1999–2000	519	57	51 (median) (4–84)	8.0 (7.3–8.7)
Ivansevic et al.^120^ (1999)	Croatia	1988–1998	278	54.4	58.3 ± 15.3 (7–89)	5.4 (4.1–6.4)
Haga et al.^117^ (2017)	Japan	2009–2011	897	62	54.4 ± 15.5 (6–95)	16.5 (15.0–18.1)
Polkinghorne et al.^125^ (2004)	New Zealand	1997–1998	146	56.7	53.9 ± 19.6 (5–96)	11.8 (9.8–13.7)
Mitry et al.^124^ (2010)	United Kingdom	2007–2009	1244	61.1	60–69 (median group)	12.1 (11.4–12.7)
Mitry et al^123^ (2011)	United Kindom	1987	–	–	–	10.1 (9.2–10.9)
		1991				11.0 (10.19–11.9)
		1996				12.5 (11.5–13.6)
		2001				12.2 (12.2–14.2)
		2006				15.28 (14.21–16.35)
Zou et al.^47^ (2002)	China	1996	61	47.5	40–59 (median group)	11.3
		1997				14.1
		1998				14.1
		1999				17.9
Burton^44^ (1989)	United States	1976	172		55.9 ± 17.9	3 (>0.00 D)
		1976				15 (–0.10 D to –6.00 D)
		1976				102 (< –6.00 D)
Chen et al.^118^ (2016)	Taiwan	2000–2012	2359	56.6	47.8 (47.1–48.4)	16.40 (15.34–17.46)

* Mean ± SD (range).

**Table 3.** Characteristics of the Studies Investigating the Relationship Between Myopia and RD

Authors	Country	Data Collection Period	Total Participants (*n*)	Study Type	Male Sex (%)	Age, y^*^	Definition of Myopia (D)	Adjusted Covariates
Ogawa and Tanaka^49^ (1988)	Japan	1961–1985	12,837	Case-control	–	–	≤ –0.75	Crude OR
Chen et al.^45^ (2018)	China	2012	749	Case-control	100	21.2 (19–25)	≤ –6.00	Crude OR
The Eye Disease Case-Control Study Group^46^ (1993)	United States	1986–1990	1,391	Case-control	47.4	– (21–80)	≤ –1.00	Crude OR
Zou et al.^47^ (2002)	China	1999	122	Case-control	–	–	<0.00	Crude OR
Chou et al.^48^ (2007)	Taiwan	1995–2001	4,569	Case-control	58.2	43 ± 18.2	≤ –1.00	Age and sex

* Mean ± SD (range).

#### Visual Burden of RD

Three studies reported BCVA after RD in myopic patients, and they all concluded that visual prognosis was often worse in myopic RD compared with nonmyopic RD.^46^^,^^50^^,^^51^ The number of patients with postoperative BCVA of <20/200 was 34% in the high myopia group (SER < –6D) compared with 19% in those without high myopia.^50^ Four studies reported on the association between myopia and reattachment of the macula after surgery. Two of these studies mentioned that reattachment of the macula after detachment was less successful in highly myopic patients, requiring more reoperations.^52^^–^^55^

### Cataract

#### Myopia and Development of Various Types of Cataract

**Figure 6.**
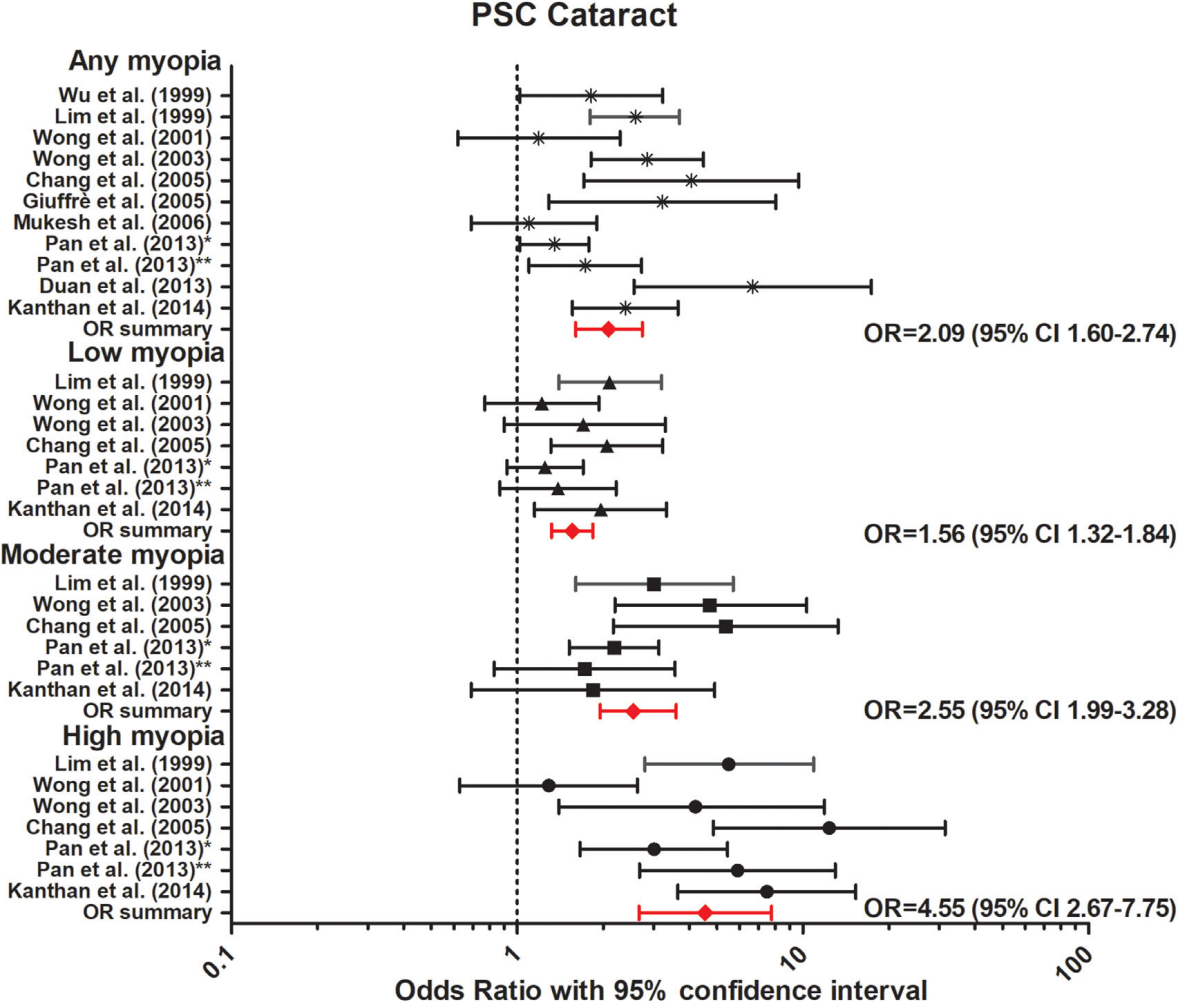
Forest plot of PSC in any myopia (random effects model; Q = 11.6; I^2^ = 13.8); low myopia (fixed effects model; Q = 7.5; I^2^ = 19.7); moderate myopia (fixed effects model; Q = 7.5; I^2^ = 19.2); and high myopia (random effects model; Q = 6.0; I^2^ = 0.14). *Red lines with diamond* represents the summary OR per myopia category, which are as follows: any myopia OR, 2.09 (95% CI, 1.60–2.74); low myopia OR, 1.56 (95% CI, 1.32–1.84); moderate myopia OR, 2.55 (95% CI, 1.99–3.28); and high myopia OR, 4.55 (95% CI, 2.67–7.75). *Represents Pan et al.^60^ 2013 Singapore Malay Eye Study. **Represents Pan et al.^61^ 2013 Singapore Indian Eye Study.

**Figure 7.**
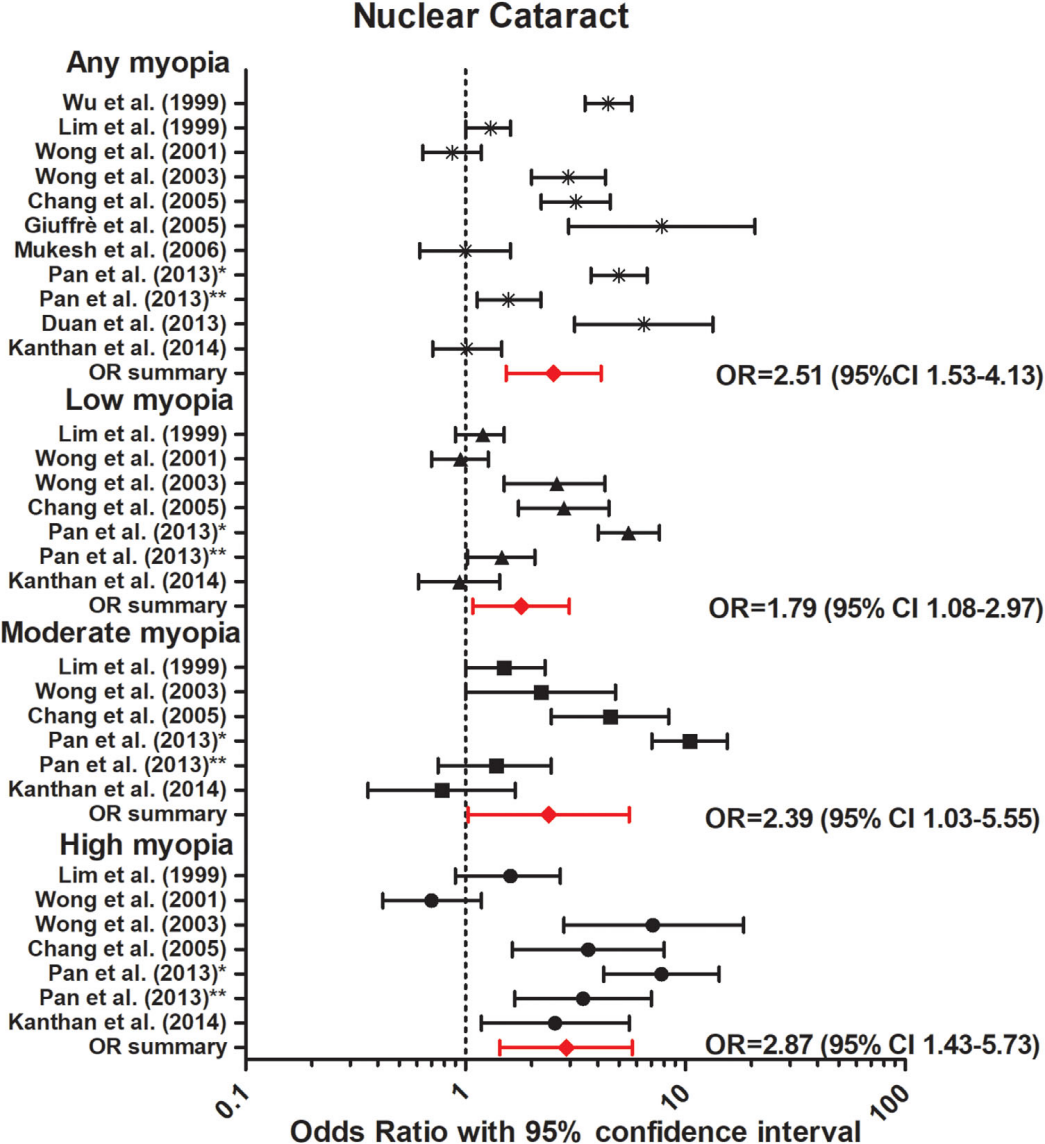
Forest plot of nuclear cataract in any myopia (random effects model; Q = 9.3; I^2^ = 0); low myopia (random effects model; Q = 5.7; I^2^ = 0); moderate myopia (random effects model; Q = 4.0; I^2^ = 0.0); and high myopia (random effects model; Q = 5.0; I^2^ = 0.0). *Red lines with diamond* represents the summary OR per myopia category, which are as follows: any myopia OR, 2.51 (95% CI, 1.53–4.13); low myopia OR, 1.79 (95% CI, 1.08–2.97); moderate myopia OR, 2.39 (95% CI, 1.03–5.55); and high myopia OR, 2.87 (95% CI, 1.43–5.73). *Represents Pan et al.^60^ 2013 Singapore Malay Eye Study. **Represents Pan et al.^61^ 2013 Singapore Indian Eye Study.

**Figure 8.**
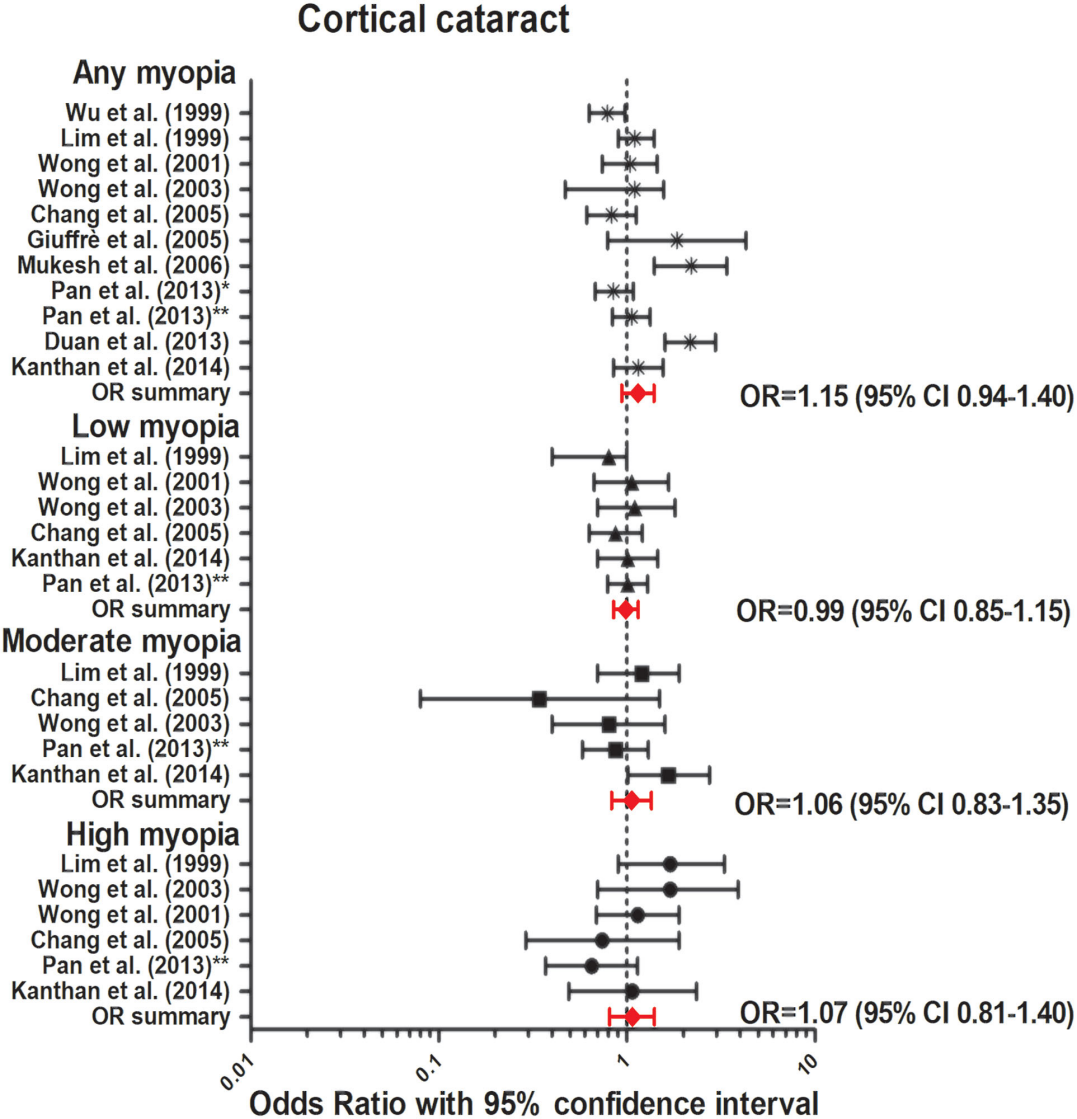
Forest plot of cortical cataract in any myopia (random effects model; Q = 11.5; I^2^ = 12.8); low myopia (fixed effects model; Q = 0.9; I^2^ = 0.0); moderate myopia (fixed effects model; Q = 7.15; I^2^ = 30.1); and high myopia (fixed effects model; Q = 6.7; I^2^ = 25.9). *Red lines with diamond* represents the summary OR per myopia category, which are as follows: any myopia OR, 1.15 (95% CI, 0.94–1.40); low myopia OR, 0.99 (95% CI, 0.85–1.15); moderate myopia OR, 1.06 (95% CI, 0.83–1.35); and high myopia OR, 1.07 (95% CI, 0.81–1.40). *Represents Pan et al.^60^ 2013 Singapore Malay Eye Study. **Represents Pan et al.^61^ 2013 Singapore Indian Eye Study.

The association between myopia and incident or prevalent cataract was investigated in three prospective and eight cross-sectional studies (Table 4).^56^^–^^66^ Nine out of 11 studies identified a strong association between myopia and posterior subcapsular cataract (PSC).^56^^–^^66^ Our meta-analysis revealed a strong association for any myopia (OR, 2.09; 95% CI, 1.60–2.74, no heterogeneity), low myopia (OR, 1.56; 95% CI, 1.32–1.84, no heterogeneity), moderate myopia (OR, 2.55; 95% CI, 1.98–3.23, no heterogeneity), and high myopia (OR, 4.55; 95% CI, 2.67–7.75, no heterogeneity) (Fig. 6). Seven out of the 11 studies reported an association between myopia and nuclear cataract, and our meta-analysis showed a significant association for any myopia (OR, 2.51; 95% CI, 1.53–4.13, no heterogeneity); low myopia (OR, 1.79; 95% CI, 1.08–2.97, no heterogeneity); moderate myopia (OR, 2.39; 95% CI, 1.03–5.55, no heterogeneity); and high myopia (OR, 2.86; 95% CI, 1.43–5.73, no heterogeneity). Regarding cortical cataract, neither prospective nor cross-sectional studies reported an association (Fig. 7). Our meta-analysis showed a summary OR of 1.15 (95% CI, 0.94–1.40, no heterogeneity) for any myopia; OR, 0.99 (95% CI, 0.85–1.15, no heterogeneity) for low myopia; OR, 1.06 (95% CI, 0.83–1.35, no heterogeneity) for moderate myopia; and OR, 1.07 (95% CI, 0.81–1.40, low heterogeneity) for high myopia (Fig. 8).

**Table 4.** Characteristics of the Studies Investigating the Relationship Between Myopia and Cataract

Study	Authors	Country	Data Collection Period	Total Participants (*n*)	Study Type	Ethnicity	Male Sex (%)	Age, y^*^	Definition of Myopia (D)	Adjusted Covariates
Blue Mountains Eye Study (BMES)	Kanthan et al.^54^ 2014	Australia	1992–2004	2564	Prospective	–	43.3	66 (49–97)	Low: –1 to ≥ –3.5 Moderate: –3.5 to ≥ –6 High: ≤ –6	Age, sex
Salisbury Eye Evaluation (SEE)	Chang et al.^58^ 2005	United States		2520	Cross-sectional	73.6% White 26.4% Black	42.1	73.0 ± 5.1	Low: –0.5 to > –4Moderate: –4 to > –6High: ≤ –6	Age, race, sex, tobacco use, education, and clustering between eyes
Beaver Dam Eye Study (BDES)	Wong et al.^57^ 2001	United States	1988–1990	3053	Prospective	–	55.1	58.8 ± 9.7	Low: –1 to –3High: ≤ –3.25	Age, sex
Blue Mountains Eye Study (BMES)	Lim et al.^59^1999	Australia	1992– 1994	3654	Cross-sectional	–	43.3	66 (49–97)	Low: –1 to > –3.5 Moderate: –3.5 > –6 High: ≤ –6	Age, sex
Singapore Malay Eye Study (SiMES)	Pan et al. 2013^60^	Singapore,	2004	3280	Cross-sectional	Malay	–	– (40–80)	Low: –0.5 to ≥ –2Moderate: –2 to ≥ –5High: < –5.0	Age, sex, body mass index, systolic blood pressure, HbA1c, smoking history, and education level
Singapore Indian Eye Study	Pan et al. 2013^61^	Singapore	2007	3400	Cross-sectional	Indian	–	– (40– 84)	Any: ≤ –0.5Low: –0.5 to > –3 High: –3 to < –6	Age, sex, smoking, education, body mass index, hypertension, and total cholesterol level
The Casteldaccia Eye Study	Giuffre et al.^65^ 2005	Italy	–	1068	Case-control	White	–	≥ 40	Any: > –1.5	None
The Barbados Eye Study	Wu et al.^66^ 1999	Barbados	1997–2003	4036	Cross-sectional	Black	43	(40–84)	Any: < –0.5	Age, sex, SES, lens opacity
The Handan Eye Study	Duan et al.^64^ 2013	China	2006–2007	6544	Cross-sectional	Chinese	46.3	52.0 ± 11.8	Any: < –0.5	Not specified (age)
The Tanjong Pagar Survey	Wong et al.^62^ 2003	Singapore	1997–1998	1029	Cross-sectional	Chinese	45.6	– (40–81)	Any: ≤ –0.5 Low: –0.5 to > –3.00 Moderate: –3.0 to > –6 High: < –6	Age, sex, education, diabetes, and smoking status
The Visual Impairment Project	Mukesh et al.^63^ 2006	Australia	1992–1999	2392	Prospective	Caucasian	45	62.5 ± 10.9	Any: < –1.0	Age, sex, country of birth, occupation, smoking status, arthritis, diabetes mellitus, vitamin C supplements, calcium channel blockers

* Mean ± SD (range). SES, socio-economic status.

#### The Risk of Cataract Extraction (CE)

**Figure 9.**
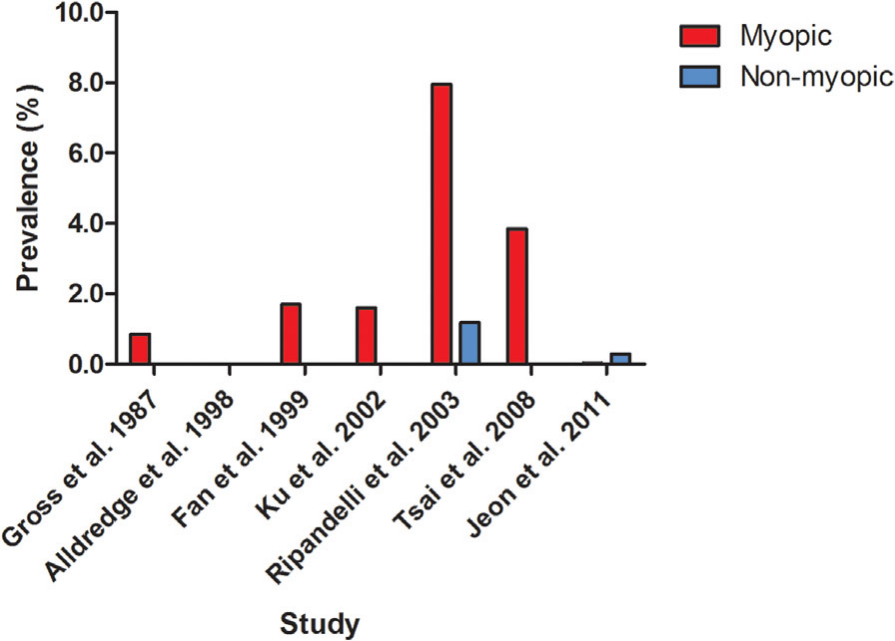
Prevalence of RD after CE in myopic patients. Horizontal axis represent different studies investigating RD rate. Two studies are case–control studies (Ripandelli et al.^73^ 2003 and Jeon et al.^72^ 2011), the other five studies are retrospective case series. The vertical axis represent the prevalence of RD.

To investigate whether CE is equally safe in myopic versus nonmyopic patients, we included seven studies investigating the association between CE in myopic patients and development of RD after CE (Fig. 9; Supplementary Table S5).^67^^–^^73^ In five retrospective case series, prevalence of RD in myopic patients ranged from 0% to 3.84%.^67^^–^^71^ Two case–control studies and one cohort study reported a significant risk of RD after CE in myopic patients (1.27% vs. 0.28%, *P* < 0.001; 8.0% vs. 1.2%, *P* < 0.01, and HR, 6.12; 95% CI, 5.84–6.41), and the association was stronger in patients undergoing CE aged younger than 55 years (HR, 25.05; 95% CI, 24.76–25.18).^72^^–^^74^ The presence of posterior vitreous detachment prior to CE was not reported.^67^^–^^71^^,^^73^^,^^74^

### Open Angle Glaucoma 

#### The Association Between Myopia and OAG

**Figure 10.**
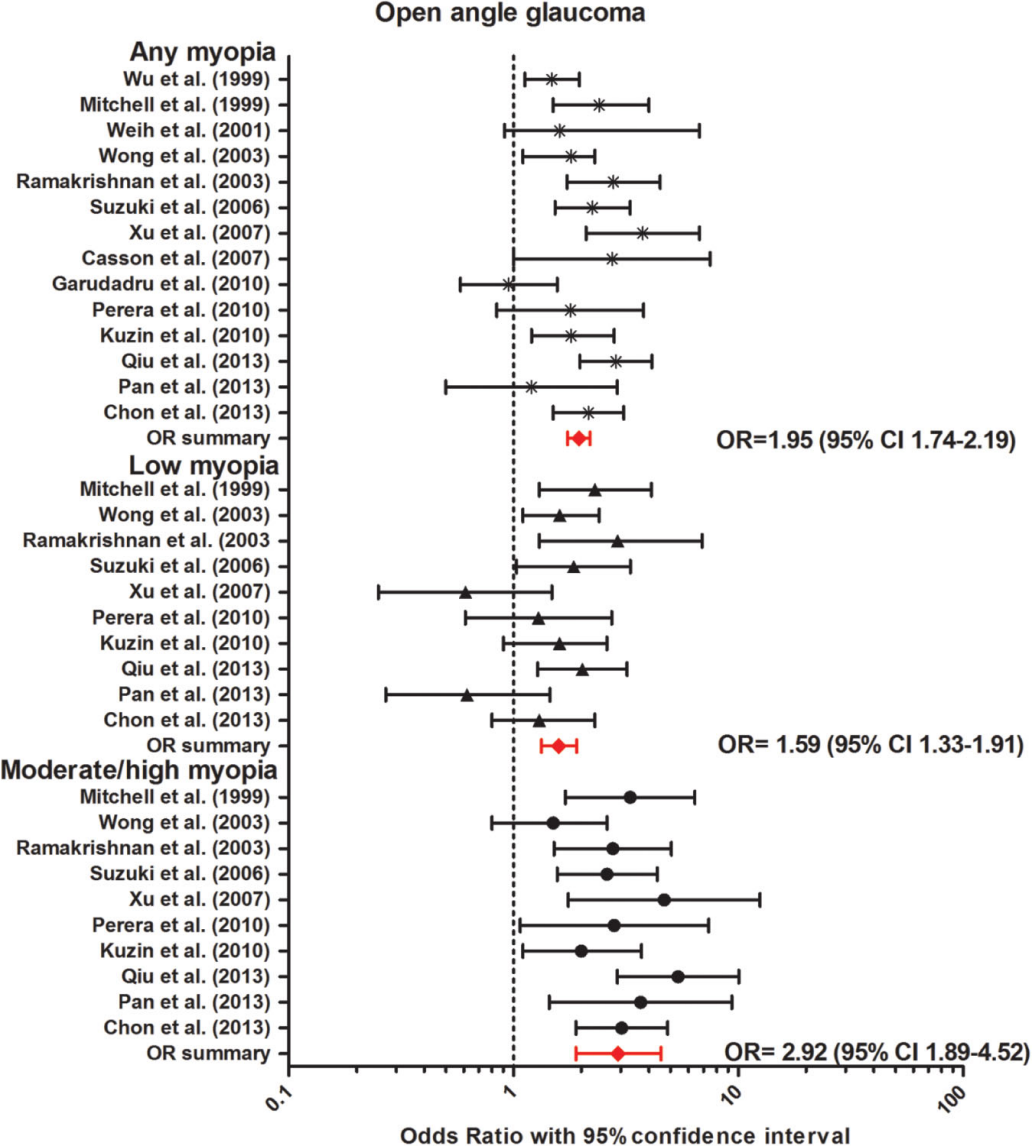
Forest plot of OAG in any myopia (fixed effects model; Q = 8.3; I^2^ = 0.0); low myopia (fixed effects model; Q = 0.3; I^2^ = 0.0); and moderate/high myopia (random effects model; Q = 2.6; I^2^ = 0.0). *Red lines with diamond* represents the summary OR per myopia category, which are as follows: any myopia OR, 1.95 (95% CI, 1.74–2.19); low myopia OR, 1.59 (95% CI, 1.33–1.91); moderate/high myopia OR, 2.92 (95% CI, 1.89–4.52).

We performed a meta-analysis of 14 population-based studies on the association between myopia and OAG (Table 5).^61^^,^^66^^,^^75^^–^^86^ Diagnosis of OAG was based on visual field defects and optic disc aberrations in most studies. The overall OR was 1.95 (95% CI, 1.74–2.19, no heterogeneity) for any myopia compared with emmetropia. The association became stronger with increasing myopia degree; the overall pooled OR was 1.59 (95% CI, 1.33–1.91, no heterogeneity) for low myopia (> –3 D); and OR, 2.92 (95% CI, 1.89–4.52, no heterogeneity) for moderate/high myopia (≤ –3 D) (Fig. 10).

**Table 5.** Characteristics of the Studies Investigating the Relationship Between Myopia and OAG

Study	Authors	Data Collection Period	Total Participants (*n*)	Study Type	Ethnicity	Age, Y	Glaucoma Definition	Definition of Myopia (D)	Adjusted Covariates
The Barbados Eye Study	Wu et al.^66^ 1999	1997–2003	4,036	Cross-sectional	Black	40–84	GVFL, optic disc abnormalities	Any: < –0.5	Age, sex, SES, lens opacity
The Blue Mountains Eye Study	Mitchell et al.^75^ 1999	1992–1994	3,654	Cross-sectional	White	49–97	GVFL, CD-ratio ≥0.7 or asymmetry ≥0.3	Any: ≤ –1.0Low: ≤1.0 to > –3.0High: ≤ –3.0	Age, sex, family history, DM, steroid use, typical migraine history, hypertension, pseudoexfoliation
Visual Impairment Project	Weih et al.^76^ 2001	1992–1996	4,498	Cross-sectional	Diverse	≥40	IOP ≥22 mm Hg, GVFL, CD-ratio ≥0.7 or asymmetry ≥0.3, family history of glaucoma	Any: ≤ –0.5	Age, rural residence, and family history
The Beaver Dam Eye Study	Wong et al.^77^ 2003	1987–1988	4,670	Cross-sectional	White	43–86	GVFL, IOP ≥22 mm Hg, CD-ratio ≥0.8 or asymmetry ≥0.2, history of glaucoma treatment	Any: ≤ –1.0Low: ≤1.0 to > –3.0High: ≤ –3.0	Age, sex
The Aravind Comprehensive Eye Survey	Ramakrishnan et al.^78^ 2003	1995–1997	5,150	Cross-sectional	Indian	≥40	GVFL, CD-ratio ≥0.9 or asymmetry ≥0.3, optic disc abnormalities, normal gonioscopy	Any: ≤-0.5Low, moderate, and high(no specific definition)	Age, sex, DM, hypertension, pseudoexfoliation
The Tajimi Study	Suzuki et al.^79^ 2006	2000–2001	2,874	Cross-sectional	Japanese	≥40	Optic disc abnormalities, perimetric results, other ocular findings	Any: ≤ –1.0 Low: ≤1.0 to > –3.0High: ≤ –3.0	Age, IOP
The Beijing Eye Study	Xu et al.^80^ 2007	2001	4,319	Cross-sectional	Chinese	≥40	Optic disc abnormalities, GVFL	Any: < –0.5 Low: <0.5 to > –3High: (< –8)	Age, IOP
The Meiktila Eye Study	Casson et al.^81^ 2007	2005	1,997	Cross-sectional	Diverse	≥40	CD-ratio ≥0.7 or ≥0.6 with asymmetry ≥0.3, reduced NRRW, GVFL, >900 of TM visible	Any: < –0.5	Age, IOP, AL
The Andhra Pradesh Eye Disease Study	Garudadri et al.^82^ 2010	1996–2000	3,724	Cross-sectional	Indian	≥40	Asymmetrical CD-ratio, NRRW reduced to 0.1, GVFL	Any: < –0.5	Age, DM, sex, IOP, hypertension
The Singapore Malay Eye Study	Perera et al.^83^ 2010	2010–2013	3,109	Cross-sectional	Malay	40–80	Optic disc abnormalities, GVFL	Any: ≤ –1.0;Low: ≤ –1.0 to >–4.0High: ≤ –4.0	Age, sex, IOP, education, height, CCT, hypertension, HbA1c
The Los Angeles Latino Eye Study	Kuzin et al.^84^ 2010	2000–2003	5,927	Cross-sectional	Latino	≥40	Optic disc abnormalities, GVFL	Any: ≤ –1.0Low: ≤1.0 to > –3.0High: ≤ –3.0	Age, IOP, DM, sex, family history, NO, CP
National Health and Nutrition Examination Survey	Qiu et al.^85^ 2013	2005–2008	5,277	Cross-sectional	Diverse	≥40	GVFL	Any: ≤ –1.0Low: –1.00 to –2.99High: ≤ –3.0	Age, sex, ethnicity, income, and education
Singapore Indian Eye Study	Pan et al.^61^ 2013	2007	3,400	Cross-sectional	Indian	40–84	Optic disc abnormalities, GVFL	Any: ≤ –0.5Low: –0.5 to –2.99High: ≤ –3.0	Age, sex, education,HbA1c, total cholesterol level, IOP, and central corneal thickness in generalized estimating equation models
Korean National Health and Nutrition Examination Survey	Chon et al.^86^ 2013	2008–2011	13,433	Cross-sectional	Korean	≥40	Optic disc abnormalities (CD-ratio ≥0.9),GVFL, or IOP >21 mm Hg and VA <3/60	Any: ≤ –1.0Low: –1.0 to –2.99High: ≤ –3.0	Age, sex, income, and education

CCT, central corneal thickness; CD, cup disc; CP, corneal power; DM, diabetes mellitus. NO, lens nuclear opacification; SES, socio-economic status; TM, trabecular meshwork.

#### Visual Burden of OAG

**Figure 11.**
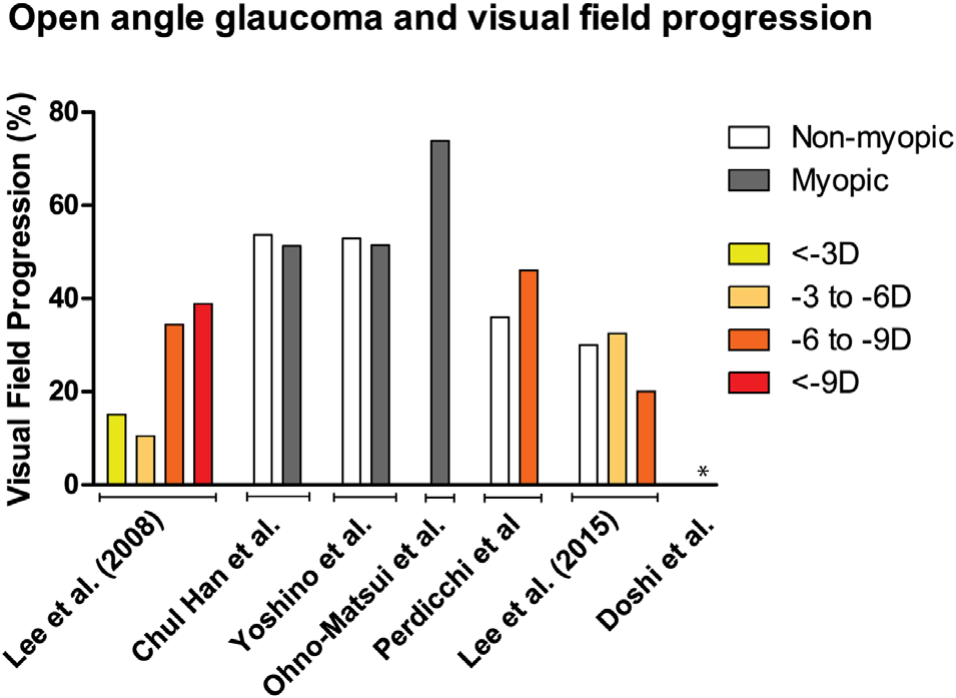
Overview of visual field progression (%) between nonmyopic and myopic patients. Different refractive error categories were indicated by *orange patterns*. Patients were categorized as myopic if refractive error category was unavailable. Doshi et al.^90^ found 0% progression in the group SER ≤ –6 D.

Seven retrospective studies, four case only, and three case–control studies reported on the association between myopia and visual field loss progression (Fig. 11; Supplementary Table S6). OAG patients with normotensive intraocular pressure under treatment were included in all studies, and follow-up length ranged from 2 to 10 years. Myopia was identified as a risk factor for visual field progression in OAG in three studies.^87^^–^^89^ However, the other four studies did not report an association.^90^^–^^93^ Whether progressive OAG is an important cause of myopic visual morbidity therefore remains questionable. Lack of data hampered investigation of the association between myopia and VA in OAG patients.

## Visual Burden of Myopia

**Figure 12.**
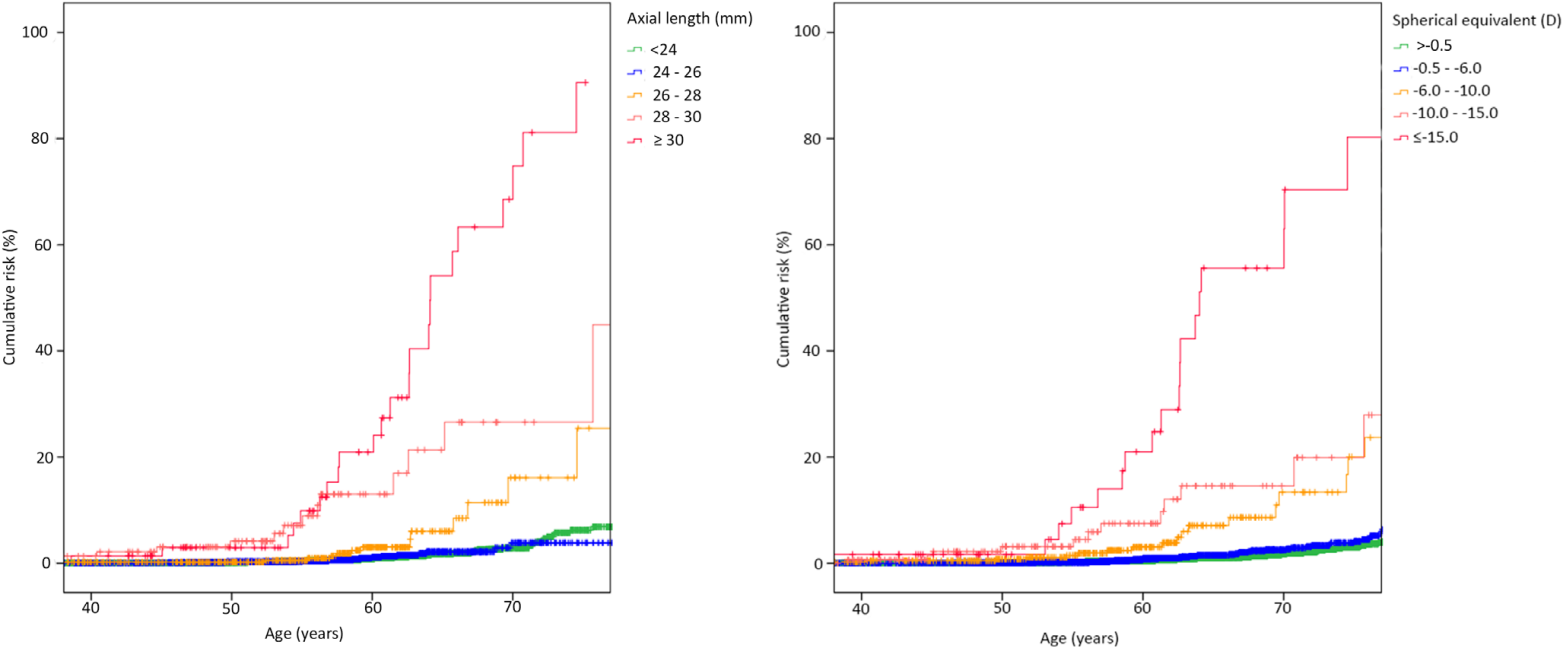
Kaplan–Meier curve of the cumulative risk of visual impairment with increasing age per category of AL (*left*) and SER (*right*). Reproduced with permission from Tideman JL, Snabel MC, Tedja MS, et al. Association of axial length with risk of uncorrectable visual impairment for Europeans with myopia. *JAMA Ophthalmol*. 2016;134:1355–1363. © 2016 American Medical Association.

Vision loss from any cause in myopia was investigated in only a few studies. A study using data from the Rotterdam Study, performed in The Netherlands, showed that 34.6% of the high myopes will eventually develop bilateral visual impairment (25.0%) or blindness (9.6%).^5^ Visual impairment (VA <0.3 and VA ≥0.05) and blindness (VA <0.05) were defined according to the World Health Organization criteria in this study.^5^ The risk of visual impairment in high myopia started to increase already before the age of 60 years.^5^ Another Dutch study, including population-based, family-based, and case–control data, investigated the association between myopia, AL, and visual impairment. An overall risk of visual impairment was reported, which increased myopia degree (OR, 0.92, 95% CI, 0.62–1.35 for SER –0.5 to > –3 D; OR, 1.71, 95% CI, 1.07–2.74 for SER –3 to > –6 D; OR, 5.54, 95% CI, 3.12–9.85 for SER –6 to > –10D; OR, 7.77, 95% CI, 3.36–17.99 for SER –10 to > –15 D; OR, 87.63, 95% CI, 34.50–222.58 for SER < –15 D in participants aged >60 years).^4^ AL was a stronger predictor for visual impairment or blindness than refractive error. The cumulative risk of visual impairment or blindness increased from 6.9% in eyes less than 24 mm, up to 90.6% in eyes of 30 mm or greater in participants aged 75 years or older.^4^ For those with AL ≥26 mm, one in three was at risk of developing bilateral low vision with increasing age. The rise in cumulative risk started at age 55 years for participants with SER ≤ –10 D, and at age 65 years for participants with SER –6 D to –10 D, and showed an almost exponential increase for SER ≤ –10D thereafter (Fig. 12).^4^ Considering visual function, 10 studies reported on ERG responses (multifocal and full-field ERG) in mostly healthy adults with different ALs, and identified decreased amplitudes of both a- and b-wave responses, correlating negatively with AL.^94^^–^^103^ Contrast sensitivity was only investigated in healthy myopic participants, and multiple studies reported a decreased contrast sensitivity in myopic compared with emmetropic participants.^104^^–^^106^

## Discussion

Our study showed that myopia is associated with MMD, RD, PSC, and OAG. The risk of these complications was not only increased for high myopia, but also for low or moderate myopia. Overall, myopic patients had 100-fold higher risk of MMD, three-fold higher risk of RD, three-fold higher risk of PSC, and an almost doubled risk of OAG.

MMD was by far the most hazardous complication. Emmetropic eyes, which served as the reference, did not develop MMD, which hampered interpretation of the high-risk estimates for myopes. Frequency data on MMD could be more informative, but nonuniform definitions, highly variable age distributions of study participants, and the potential selection bias due to hospital recruitment caused large heterogeneity in prevalence estimates. MMD prevalence ranged from 0.1% to 7% in low myopia, 0.3% to 10% in moderate myopia, and 13% to 65% in high myopia.^24^^–^^26^^,^^29^ BCVA was generally worse in patients with macular atrophy, CNV, or Fuchs spot.^23^^–^^25^^,^^36^^,^^41^^,^^43^ Tessellation of the fundus did not influence VA, but may increase the risk of more severe MMD with age.^42^

Our meta-analysis revealed an increased risk for RD in all myopia groups, with higher risk for those with more severe myopia. The OR for moderate myopia was already 8.7, and given the relatively high frequency of myopes in this category, the RD prevalence is expected to rise dramatically. Frequency data of RD per degree of myopia were limited in literature, but Japan and Taiwan reported remarkably higher incidence rates of RD than other countries with a lower myopia prevalence.^14^ This confirms the notion that RD rates will increase when myopia becomes more prevalent.^107^ The visual prognosis of myopic RD appeared to be worse than nonmyopic RD in some studies, but this needs more comprehensive research.^52^^–^^55^

Our meta-analysis identified a strong association between myopia, PSC, and nuclear cataract, but not between myopia and cortical cataract. Three mechanisms have been proposed to explain the relationship between myopia and cataract. First, myopic eyes may be exposed to a higher level of oxidative stress caused by faster vitreous liquefaction, or by a decreased level of glutathione, an antioxidative agent in the lens of myopic eyes leading to cataract formation.^56^^,^^108^^,^^109^ Second, the higher level of byproducts of lipid peroxidation in myopia may increase cataract formation.^56^^,^^110^^–^^112^ Third, longer AL may lead to diminished diffusion of nutrients from the posterior chamber to the lens causing cataract. This mechanism seems less plausible because the aqueous humor also provides nutrients to the lens.^58^ It should be noted that the association between myopia and nuclear cataract may be influenced by the myopic shift occurring with this type of cataract.^9^ Cataract is a disorder that can be resolved rather easily by performing CE. In myopic patients, however, reports suggest an increased risk of postsurgery RD, as CE causes a disruption of the capsular-zonular diaphragm and vitreous traction of a thin peripheral retina may then predispose to RD in myopes.^69^^,^^70^^,^^113^ However, the long interval between CE and RD in some studies makes a direct causal relationship unlikely.^72^^–^^74^ The procedure itself may be more difficult. After vitreous removal in high myopes zonular weakness may occur, leading to potential zonular instability. In addition, sculpting maneuvers may be more difficult due to a deeper anterior chamber.^114^ Given all considerations, when posterior vitreous detachment has taken place and substantial vision loss due to lens opacities is present, the visual benefits outweigh the risks and CE is recommended.^74^ Nevertheless, careful preoperative inspection for retinal tears and prophylactic treatment with laser are warranted.^67^^,^^68^^,^^73^

The positive association between myopia and OAG is in line with previous reports.^11^ Distinguishing myopic optic neuropathy from OAG remains a challenge, and may have led to misclassification and invalid estimations of the calculated OR.^115^ Considering that myopic eyes have larger optic disc sizes, and therefore larger excavations, OAG is prone to misdiagnosis. The underlying mechanism for a predisposition to OAG is still unclear. Doshi et al.^90^ mentioned that longer AL leads to tilting of the optic disc, and may possibly cause damage to the axons in the lamina cribrosa. Considering the differences in study design and definitions myopic OAG may unlikely progress to central visual field defects.

To our knowledge, this is the first systematic review and meta-analysis regarding complications associated with myopia. One of the strengths is the completeness of our literature search. We believe that we included all observational studies performed from 1988–2019 in the meta-analyses. Another asset is the estimations of risk per refractive error category, which elucidated the profound risk increase for the higher degrees of myopia, but also revealed substantial risks for the much more common low and moderate myopia. Limitations of our study include the different definitions used for myopic complications, in particular for MMD and OAG. We strived to use the recently defined guidelines by the International Myopia Institute to optimize uniformity between studies, but sometimes had to apply best clinical judgement if this was not possible.^20^ Our decisions may have affected the results. Another limitation was the lack of multimodal imaging to detect all retinal complications; most studies only used color fundus photographs. In particular, retinoschisis, macular hole, different types of staphylomas, and peripheral lesions are better visualized with other imaging techniques, such as optical coherence tomography and wide-field imaging. We therefore chose to focus only on MMD, RD, cataract, and OAG. We expect that future studies will provide more results using newer and multimodal imaging techniques. Finally, although AL is more closely related to myopic complications than refractive error, we could not study this for most complications, as data on eye biometry were missing.

Regarding clinical management, the results from our meta-analyses suggest that vision-threatening complications can appear from moderate myopia onward. There is a strong relationship between myopia degree, age of the participant, and visual impairment; more severe myopia results in an earlier onset of visual-threatening complications.^4^^,^^5^ Therefore both factors should be taken into account regarding screening programs and clinical guidelines. A period of 20 years between diagnosis and perimetric blindness was estimated for OAG patients with average visual field loss progression.^116^^,^^117^ A significant visual loss over a follow-up period of 10 years was determined for the natural course of MMD.^40^^,^^42^ Considering the asymptomatic period and window of possible action before the onset of complications, we advise an ophthalmologic screen at the age of 30 in myopic patients with SER ≤ –10 D, and at the age of 50 in patients with SER –6 D to –10 D.

## Conclusions

This literature review and meta-analyses provide detailed risk estimates of myopic complications. One in three high myopes is at risk of bilateral low vision with age. Low and moderate myopes are less likely to develop such a severe visual outcome; nevertheless, they are at significant risk to develop MMD, RD, cataract, and OAG. This not only affects the individual patient, it has a major impact on health care and society, in particular because future generations may become even more myopic. Awareness of the complications of myopia among patients, physicians, and policy makers is crucial, and a global strategy for prevention and treatment of myopia progression should become a priority.

## Supplementary Material

Supplement 1

Supplement 2

## References

[1] Morgan I, Rose K How genetic is school myopia? *Prog Retin Eye Res*. 2005; 24: 1–38. 15555525 10.1016/j.preteyeres.2004.06.004

[2] Meng W, Butterworth J, Malecaze F, Calvas P Axial length of myopia: a review of current research. *Ophthalmologica*. 2011; 225: 127–134. 20948239 10.1159/000317072

[3] Flitcroft DI The complex interactions of retinal, optical and environmental factors in myopia aetiology. *Prog Retin Eye Res*. 2012; 31: 622–660. 22772022 10.1016/j.preteyeres.2012.06.004

[4] Tideman JL, Snabel MC, Tedja MS, et al. Association of axial length with risk of uncorrectable visual impairment for Europeans with myopia. *JAMA Ophthalmol*. 2016; 134: 1355–1363. 27768171 10.1001/jamaophthalmol.2016.4009

[5] Verhoeven VJ, Wong KT, Buitendijk GH, Hofman A, Vingerling JR, Klaver CC Visual consequences of refractive errors in the general population. *Ophthalmology*. 2015; 122: 101–109. 25208857 10.1016/j.ophtha.2014.07.030

[6] Ohno-Matsui K, Kawasaki R, Jonas JB, et al. International photographic classification and grading system for myopic maculopathy. *Am J Ophthalmol*. 2015; 159: 877–883.e7. 25634530 10.1016/j.ajo.2015.01.022

[7] Avila MP, Weiter JJ, Jalkh AE, Trempe CL, Pruett RC, Schepens CL Natural history of choroidal neovascularization in degenerative myopia. *Ophthalmology*. 1984; 91: 1573–1581. 6084222 10.1016/s0161-6420(84)34116-1

[8] Lam DS, Fan DS, Chan WM, et al. Prevalence and characteristics of peripheral retinal degeneration in Chinese adults with high myopia: a cross-sectional prevalence survey. *Optom Vis Sci*. 2005; 82: 235–238. 15829850 10.1097/01.opx.0000159359.49457.b4

[9] Brown NA, Hill AR Cataract: the relation between myopia and cataract morphology. *Br J Ophthalmol*. 1987; 71: 405–414. 3620419 10.1136/bjo.71.6.405 PMC1041188

[10] Perkins ES, Phelps CD Open angle glaucoma, ocular hypertension, low-tension glaucoma, and refraction. *Arch Ophthalmol*. 1982; 100: 1464–1467. 7115175 10.1001/archopht.1982.01030040442015

[11] Marcus MW, de Vries MM, Junoy Montolio FG, Jansonius NM Myopia as a risk factor for open-angle glaucoma: a systematic review and meta-analysis. *Ophthalmology*. 2011; 118: 1989–1994.e2. 21684603 10.1016/j.ophtha.2011.03.012

[12] Rose K, Harper R, Tromans C, et al. Quality of life in myopia. *Br J Ophthalmol*. 2000; 84: 1031–1034. 10966960 10.1136/bjo.84.9.1031 PMC1723631

[13] Holden B, Sankaridurg P, Smith E, Aller T, Jong M, He M Myopia, an underrated global challenge to vision: where the current data takes us on myopia control. *Eye (Lond)*. 2014; 28: 142–146. 24357836 10.1038/eye.2013.256 PMC3930268

[14] Holden BA, Fricke TR, Wilson DA, et al. Global prevalence of myopia and high myopia and temporal trends from 2000 through 2050. *Ophthalmology*. 2016; 123: 1036–1042. 26875007 10.1016/j.ophtha.2016.01.006

[15] Naidoo KS, Fricke TR, Frick KD, et al. Potential lost productivity resulting from the global burden of myopia: systematic review, meta-analysis, and modeling. *Ophthalmology*. 2019; 126: 338–346. 30342076 10.1016/j.ophtha.2018.10.029

[16] Fricke TR, Jong M, Naidoo KS, et al. Global prevalence of visual impairment associated with myopic macular degeneration and temporal trends from 2000 through 2050: systematic review, meta-analysis and modelling. *Br J Ophthalmol*. 2018; 102: 855–862. 29699985 10.1136/bjophthalmol-2017-311266 PMC6047154

[17] Morgan IG, Ohno-Matsui K, Saw S-M Myopia. *Lancet*. 2012; 379: 1739–1748. 22559900 10.1016/S0140-6736(12)60272-4

[18] Moher D, Liberati A, Tetzlaff J, Altman DG, Group P Preferred reporting items for systematic reviews and meta-analyses: the PRISMA statement. *PLoS Med*. 2009; 6: e1000097. 19621072 10.1371/journal.pmed.1000097 PMC2707599

[19] Sanderson S, Tatt ID, Higgins J Tools for assessing quality and susceptibility to bias in observational studies in epidemiology: a systematic review and annotated bibliography. *Int J Epidemiol*. 2007; 36: 666–676. 17470488 10.1093/ije/dym018

[20] Flitcroft DI, He M, Jonas JB, et al. IMI-defining and classifying myopia: a proposed set of standards for clinical and epidemiologic studies. *Invest Ophthalmol Vis Sci*. 2019; 60: M20–M30. 30817826 10.1167/iovs.18-25957 PMC6735818

[21] Neyeloff JL, Fuchs SC, Moreira LB Meta-analyses and Forest plots using a Microsoft Excel spreadsheet: step-by-step guide focusing on descriptive data analysis. *BMC Res Notes*. 2012; 5: 52. 22264277 10.1186/1756-0500-5-52 PMC3296675

[22] Higgins JPT, Thompson SG, Deeks JJ, Altman DG Measuring inconsistency in meta-analyses. *BMJ*. 2003; 327: 557–560. 12958120 10.1136/bmj.327.7414.557 PMC192859

[23] Vongphanit J, Mitchell P, Wang JJ Prevalence and progression of myopic retinopathy in an older population. *Ophthalmology*. 2002; 109: 704–711. 11927427 10.1016/s0161-6420(01)01024-7

[24] Liu HH, Xu L, Wang YX, Wang S, You QS, Jonas JB Prevalence and progression of myopic retinopathy in Chinese adults: the Beijing Eye Study. *Ophthalmology*. 2010; 117: 1763–1768. 20447693 10.1016/j.ophtha.2010.01.020

[25] Gao LQ, Liu W, Liang YB, et al. Prevalence and characteristics of myopic retinopathy in a rural Chinese adult population: the Handan Eye Study. *Arch Ophthalmol*. 2011; 129: 1199–1204. 21911668 10.1001/archophthalmol.2011.230

[26] Asakuma T, Yasuda M, Ninomiya T, et al. Prevalence and risk factors for myopic retinopathy in a Japanese population: the Hisayama Study. *Ophthalmology*. 2012; 119: 1760–1765. 22578442 10.1016/j.ophtha.2012.02.034

[27] Jonas JB, Nangia V, Gupta R, Bhojwani K, Nangia P, Panda-Jonas S Prevalence of myopic retinopathy in rural central India. *Acta Ophthalmol*. 2017; 95: e399–e404. 27860316 10.1111/aos.13301

[28] Chen SJ, Cheng CY, Li AF, et al. Prevalence and associated risk factors of myopic maculopathy in elderly Chinese: the Shihpai eye study. *Invest Ophthalmol Vis Sci*. 2012; 53: 4868–4873. 22743322 10.1167/iovs.12-9919

[29] Wong YL, Sabanayagam C, Ding Y, et al. Prevalence, risk factors, and impact of myopic macular degeneration on visual impairment and functioning among adults in Singapore. *Invest Ophthalmol Vis Sci*. 2018; 59: 4603–4613. 30242361 10.1167/iovs.18-24032

[30] Choudhury F, Meuer SM, Klein R, et al. Prevalence and characteristics of myopic degeneration in an adult Chinese American population: the Chinese American Eye Study. *Am J Ophthalmol*. 2018; 187: 34–42. 29288031 10.1016/j.ajo.2017.12.010 PMC5837945

[31] Chen H, Wen F, Li H, et al. The types and severity of high myopic maculopathy in Chinese patients. *Ophthalmic Physiol Opt*. 2012; 32: 60–67. 21762440 10.1111/j.1475-1313.2011.00861.x

[32] Chang L, Pan C-W, Ohno-Matsui K, et al. Myopia-related fundus changes in Singapore adults with high myopia. *Am J Ophthalmol*. 2013; 155: 991–999.e1. 23499368 10.1016/j.ajo.2013.01.016

[33] Lai TYY, Fan DSP, Lai WWK, Lam DSC Peripheral and posterior pole retinal lesions in association with high myopia: a cross-sectional community-based study in Hong Kong. *Eye*. 2008; 22: 209. 16946749 10.1038/sj.eye.6702573

[34] Koh VT, Nah GK, Chang L, et al. Pathologic changes in highly myopic eyes of young males in Singapore. *Ann Acad Med Singapore*. 2013; 42: 216–224. 23771108

[35] Xiao O, Guo X, Wang D, et al. Distribution and severity of myopic maculopathy among highly myopic eyes. *Invest Ophthalmol Vis Sci*. 2018; 59: 4880–4885. 30347081 10.1167/iovs.18-24471

[36] Zhao X, Ding X, Lyu C, et al. Morphological characteristics and visual acuity of highly myopic eyes with different severities of myopic maculopathy. *Retina*. 2020; 40: 461–467. 30576301 10.1097/IAE.0000000000002418

[37] Koh V, Tan C, Tan PT, et al. Myopic maculopathy and optic disc changes in highly myopic young Asian eyes and impact on visual acuity. *Am J Ophthalmol*. 2016; 164: 69–79. 26850176 10.1016/j.ajo.2016.01.005

[38] Bikbov MM, Kazakbaeva GM, Gilmanshin TR, et al. Axial length and its associations in a Russian population: the Ural Eye and Medical Study. *PLoS One*. 2019; 14: e0211186. 30707718 10.1371/journal.pone.0211186 PMC6358075

[39] Hashimoto S, Yasuda M, Fujiwara K, et al. Association between axial length and myopic maculopathy: the Hisayama Study. *Ophthalmology Retina*. 2019; 3: 867–873. 31202664 10.1016/j.oret.2019.04.023

[40] Shih YF, Ho TC, Hsiao CK, Lin LL Visual outcomes for high myopic patients with or without myopic maculopathy: a 10 year follow up study. *Br J Ophthalmol*. 2006; 90: 546–550. 16622083 10.1136/bjo.2005.081992 PMC1857055

[41] Lichtwitz O, Boissonnot M, Mercie M, Ingrand P, Leveziel N Prevalence of macular complications associated with high myopia by multimodal imaging. *J Fr Ophtalmol*. 2016; 39: 355–363. 27016335 10.1016/j.jfo.2015.11.005

[42] Hayashi K, Ohno-Matsui K, Shimada N, et al. Long-term pattern of progression of myopic maculopathy: a natural history study. *Ophthalmology*. 2010; 117: 1595–1611.e1–4. 20207005 10.1016/j.ophtha.2009.11.003

[43] Hayasaka S, Uchida M, Setogawa T Subretinal hemorrhages with or without choroidal neovascularization in the maculas of patients with pathologic myopia. *Graefes Arch Clin Exp Ophthalmol*. 1990; 228: 277–280. 1698169 10.1007/BF00920048

[44] Burton TC The influence of refractive error and lattice degeneration on the incidence of retinal detachment. *Trans Am Ophthalmol Soc*. 1989; 87: 143–157. 2562517 PMC1298542

[45] Chen DZ, Koh V, Tan M, et al. Peripheral retinal changes in highly myopic young Asian eyes. *Acta Ophthalmol*. 2018; 96: e846–e851. 29575821 10.1111/aos.13752

[46] Risk factors for idiopathic rhegmatogenous retinal detachment. The Eye Disease Case-Control Study Group. *Am J Epidemiol*. 1993; 137: 749–757. 8484366

[47] Zou H, Zhang X, Xu X, Wang X, Liu K, Ho PC Epidemiology survey of rhegmatogenous retinal detachment in Beixinjing District, Shanghai, China. *Retina*. 2002; 22: 294–299. 12055462 10.1097/00006982-200206000-00007

[48] Chou SC, Yang CH, Lee CH, et al. Characteristics of primary rhegmatogenous retinal detachment in Taiwan. *Eye (Lond)*. 2007; 21: 1056–1061. 16691255 10.1038/sj.eye.6702397

[49] Ogawa A, Tanaka M The relationship between refractive errors and retinal detachment–analysis of 1,166 retinal detachment cases. *Jpn J Ophthalmol*. 1988; 32: 310–315. 3230716

[50] Salicone A, Smiddy WE, Venkatraman A, Feuer W Visual recovery after scleral buckling procedure for retinal detachment. *Ophthalmology*. 2006; 113: 1734–1742. 17011955 10.1016/j.ophtha.2006.03.064

[51] Ross WH, Stockl FA Visual recovery after retinal detachment. *Curr Opin Ophthalmol*. 2000; 11: 191–194. 10977226 10.1097/00055735-200006000-00007

[52] Tornquist R, Tornquist P Retinal detachment. A study of a population-based patient material in Sweden 1971-1981. III. Surgical results. *Acta Ophthalmol (Copenh)*. 1988; 66: 630–636. 3232504 10.1111/j.1755-3768.1988.tb04052.x

[53] Arias L, Caminal JM, Rubio MJ, et al. Autofluorescence and axial length as prognostic factors for outcomes of macular hole retinal detachment surgery in high myopia. *Retina*. 2015; 35: 423–428. 25170859 10.1097/IAE.0000000000000335

[54] Sharma T, Challa JK, Ravishankar KV, Murugesan R Scleral buckling for retinal detachment. Predictors for anatomic failure. *Retina*. 1994; 14: 338–343. 7817027 10.1097/00006982-199414040-00008

[55] Grizzard WS, Hilton GF, Hammer ME, Taren D A multivariate analysis of anatomic success of retinal detachments treated with scleral buckling. *Graefes Arch Clin Exp Ophthalmol*. 1994; 232: 1–7. 8119596 10.1007/BF00176431

[56] Kanthan GL, Mitchell P, Rochtchina E, Cumming RG, Wang JJ Myopia and the long-term incidence of cataract and cataract surgery: the Blue Mountains Eye Study. *Clin Exp Ophthalmol*. 2014; 42: 347–353. 24024555 10.1111/ceo.12206

[57] Wong TY, Klein BE, Klein R, Tomany SC, Lee KE Refractive errors and incident cataracts: the Beaver Dam Eye Study. *Invest Ophthalmol Vis Sci*. 2001; 42: 1449–1454. 11381046

[58] Chang MA, Congdon NG, Bykhovskaya I, Munoz B, West SK The association between myopia and various subtypes of lens opacity: SEE (Salisbury Eye Evaluation) project. *Ophthalmology*. 2005; 112: 1395–1401. 15953641 10.1016/j.ophtha.2005.02.017

[59] Lim R, Mitchell P, Cumming RG Refractive associations with cataract: the Blue Mountains Eye Study. *Invest Ophthalmol Vis Sci*. 1999; 40: 3021–3026. 10549667

[60] Pan CW, Boey PY, Cheng CY, et al. Myopia, axial length, and age-related cataract: the Singapore Malay eye study. *Invest Ophthalmol Vis Sci*. 2013; 54: 4498–4502. 23737473 10.1167/iovs.13-12271

[61] Pan CW, Cheung CY, Aung T, et al. Differential associations of myopia with major age-related eye diseases: the Singapore Indian Eye Study. *Ophthalmology*. 2013; 120: 284–291. 23084122 10.1016/j.ophtha.2012.07.065

[62] Wong TY, Foster PJ, Johnson GJ, Seah SK Refractive errors, axial ocular dimensions, and age-related cataracts: the Tanjong Pagar survey. *Invest Ophthalmol Vis Sci*. 2003; 44: 1479–1485. 12657582 10.1167/iovs.02-0526

[63] Mukesh BN, Le A, Dimitrov PN, Ahmed S, Taylor HR, McCarty CA Development of cataract and associated risk factors: the Visual Impairment Project. *Arch Ophthalmol*. 2006; 124: 79–85. 16401788 10.1001/archopht.124.1.79

[64] Duan XR, Liang YB, Wang NL, et al. Prevalence and associations of cataract in a rural Chinese adult population: the Handan Eye Study. *Graefes Arch Clin Exp Ophthalmol*. 2013; 251: 203–212. 22527317 10.1007/s00417-012-2012-x

[65] Giuffre G, Dardanoni G, Lodato G A case-control study on risk factors for nuclear, cortical and posterior subcapsular cataract: the Casteldaccia Eye Study. *Acta Ophthalmol Scand*. 2005; 83: 567–573. 16187994 10.1111/j.1600-0420.2005.00475.x

[66] Wu SY, Nemesure B, Leske MC Refractive errors in a black adult population: the Barbados Eye Study. *Invest Ophthalmol Vis Sci*. 1999; 40: 2179–2184. 10476781

[67] Fan DS, Lam DS, Li KK Retinal complications after cataract extraction in patients with high myopia. *Ophthalmology*. 1999; 106: 688–691; discussion 691–682. 10201588 10.1016/S0161-6420(99)90152-5

[68] Tsai CY, Chang TJ, Kuo LL, Chou P, Woung LC Visual outcomes and associated risk factors of cataract surgeries in highly myopic Taiwanese. *Ophthalmologica*. 2008; 222: 130–135. 18303235 10.1159/000112631

[69] Ku WC, Chuang LH, Lai CC Cataract extraction in high myopic eyes. *Chang Gung Med J*. 2002; 25: 315–320. 12141704

[70] Alldredge CD, Elkins B, Alldredge OC Jr. Retinal detachment following phacoemulsification in highly myopic cataract patients. *J Cataract Refract Surg*. 1998; 24: 777–780. 9642587 10.1016/s0886-3350(98)80130-2

[71] Gross KA, Pearce JL Modern cataract surgery in a highly myopic population. *Br J Ophthalmol*. 1987; 71: 215–219. 3828280 10.1136/bjo.71.3.215 PMC1041123

[72] Jeon S, Kim HS Clinical characteristics and outcomes of cataract surgery in highly myopic Koreans. *Korean J Ophthalmol*. 2011; 25: 84–89. 21461219 10.3341/kjo.2011.25.2.84 PMC3060398

[73] Ripandelli G, Scassa C, Parisi V, Gazzaniga D, D'Amico DJ, Stirpe M Cataract surgery as a risk factor for retinal detachment in very highly myopic eyes. *Ophthalmology*. 2003; 110: 2355–2361. 14644718 10.1016/S0161-6420(03)00819-4

[74] Daien V, Le Pape A, Heve D, Carriere I, Villain M Incidence, risk factors, and impact of age on retinal detachment after cataract surgery in France: a national population study. *Ophthalmology*. 2015; 122: 2179–2185. 26278859 10.1016/j.ophtha.2015.07.014

[75] Mitchell P, Hourihan F, Sandbach J, Wang JJ The relationship between glaucoma and myopia: the Blue Mountains Eye Study. *Ophthalmology*. 1999; 106: 2010–2015. 10519600 10.1016/s0161-6420(99)90416-5

[76] Weih LM, Nanjan M, McCarty CA, Taylor HR Prevalence and predictors of open-angle glaucoma: results from the visual impairment project. *Ophthalmology*. 2001; 108: 1966–1972. 11713063 10.1016/s0161-6420(01)00799-0

[77] Wong TY, Klein BE, Klein R, Knudtson M, Lee KE Refractive errors, intraocular pressure, and glaucoma in a white population. *Ophthalmology*. 2003; 110: 211–217. 12511368 10.1016/s0161-6420(02)01260-5

[78] Ramakrishnan R, Nirmalan PK, Krishnadas R, et al. Glaucoma in a rural population of southern India: the Aravind comprehensive eye survey. *Ophthalmology*. 2003; 110: 1484–1490. 12917161 10.1016/S0161-6420(03)00564-5

[79] Suzuki Y, Iwase A, Araie M, et al. Risk factors for open-angle glaucoma in a Japanese population: the Tajimi Study. *Ophthalmology*. 2006; 113: 1613–1617. 16828504 10.1016/j.ophtha.2006.03.059

[80] Xu L, Wang Y, Wang S, Wang Y, Jonas JB High myopia and glaucoma susceptibility the Beijing Eye Study. *Ophthalmology*. 2007; 114: 216–220. 17123613 10.1016/j.ophtha.2006.06.050

[81] Casson RJ, Gupta A, Newland HS, et al. Risk factors for primary open-angle glaucoma in a Burmese population: the Meiktila Eye Study. *Clin Exp Ophthalmol*. 2007; 35: 739–744. 17997778 10.1111/j.1442-9071.2007.01619.x

[82] Garudadri C, Senthil S, Khanna RC, Sannapaneni K, Rao HB Prevalence and risk factors for primary glaucomas in adult urban and rural populations in the Andhra Pradesh Eye Disease Study. *Ophthalmology*. 2010; 117: 1352–1359. 20188420 10.1016/j.ophtha.2009.11.006

[83] Perera SA, Wong TY, Tay WT, Foster PJ, Saw SM, Aung T Refractive error, axial dimensions, and primary open-angle glaucoma: the Singapore Malay Eye Study. *Arch Ophthalmol*. 2010; 128: 900–905. 20625053 10.1001/archophthalmol.2010.125

[84] Kuzin AA, Varma R, Reddy HS, Torres M, Azen SP; Los Angeles Latino Eye Study Group. Ocular biometry and open-angle glaucoma: the Los Angeles Latino Eye Study. *Ophthalmology*. 2010; 117: 1713–1719. 20570359 10.1016/j.ophtha.2010.01.035 PMC2934756

[85] Qiu M, Wang SY, Singh K, Lin SC Association between myopia and glaucoma in the United States population. *Invest Ophthalmol Vis Sci*. 2013; 54: 830–835. 23299483 10.1167/iovs.12-11158 PMC3562121

[86] Chon B, Qiu M, Lin SC Myopia and glaucoma in the South Korean population. *Invest Ophthalmol Vis Sci*. 2013; 54: 6570–6577. 24022009 10.1167/iovs.13-12173

[87] Ohno-Matsui K, Shimada N, Yasuzumi K, et al. Long-term development of significant visual field defects in highly myopic eyes. *Am J Ophthalmol*. 2011; 152: 256–265.e1. 21664594 10.1016/j.ajo.2011.01.052

[88] Perdicchi A, Iester M, Scuderi G, Amodeo S, Medori EM, Recupero SM Visual field damage and progression in glaucomatous myopic eyes. *Eur J Ophthalmol*. 2007; 17: 534–537. 17671927 10.1177/112067210701700409

[89] Lee YA, Shih YF, Lin LL, Huang JY, Wang TH Association between high myopia and progression of visual field loss in primary open-angle glaucoma. *J Formos Med Assoc*. 2008; 107: 952–957. 19129056 10.1016/S0929-6646(09)60019-X

[90] Doshi A, Kreidl KO, Lombardi L, Sakamoto DK, Singh K Nonprogressive glaucomatous cupping and visual field abnormalities in young Chinese males. *Ophthalmology*. 2007; 114: 472–479. 17123617 10.1016/j.ophtha.2006.07.036

[91] Han JC, Lee EJ, Kim SH, Kee C Visual field progression pattern associated with optic disc tilt morphology in myopic open-angle glaucoma. *Am J Ophthalmol*. 2016; 169: 33–45. 27318077 10.1016/j.ajo.2016.06.005

[92] Yoshino T, Fukuchi T, Togano T, et al. Rate of progression of total, upper, and lower visual field defects in patients with open-angle glaucoma and high myopia. *Jpn J Ophthalmol*. 2016; 60: 78–85. 26822678 10.1007/s10384-016-0427-3

[93] Lee JY, Sung KR, Han S, Na JH Effect of myopia on the progression of primary open-angle glaucoma. *Invest Ophthalmol Vis Sci*. 2015; 56: 1775–1781. 25698704 10.1167/iovs.14-16002

[94] Kawabata H, Adachi-Usami E Multifocal electroretinogram in myopia. *Invest Ophthalmol Vis Sci*. 1997; 38: 2844–2851. 9418738

[95] Westall CA, Dhaliwal HS, Panton CM, et al. Values of electroretinogram responses according to axial length. *Doc Ophthalmol*. 2001; 102: 115–130. 11518455 10.1023/a:1017535207481

[96] Hidajat R, McLay J, Burley C, Elder M, Morton J, Goode D Influence of axial length of normal eyes on PERG. *Doc Ophthalmol*. 2003; 107: 195–200. 14661910 10.1023/a:1026282425885

[97] Luu CD, Lau AM, Lee SY Multifocal electroretinogram in adults and children with myopia. *Arch Ophthalmol*. 2006; 124: 328–334. 16534052 10.1001/archopht.124.3.328

[98] Kader MA Electrophysiological study of myopia. *Saudi J Ophthalmol*. 2012; 26: 91–99. 23960975 10.1016/j.sjopt.2011.08.002 PMC3729644

[99] Ho WC, Kee CS, Chan HH Myopic children have central reduction in high contrast multifocal ERG response, while adults have paracentral reduction in low contrast response. *Invest Ophthalmol Vis Sci*. 2012; 53: 3695–3702. 22570348 10.1167/iovs.11-9379

[100] Koh V, Tan C, Nah G, et al. Correlation of structural and electrophysiological changes in the retina of young high myopes. *Ophthalmic Physiol Opt*. 2014; 34: 658–666. 25331579 10.1111/opo.12159

[101] Ismael ZF, El-Shazly AAE, Farweez YA, Osman MMM Relationship between functional and structural retinal changes in myopic eyes. *Clin Exp Optom*. 2017; 100: 695–703. 28226407 10.1111/cxo.12527

[102] Sachidanandam R, Ravi P, Sen P Effect of axial length on full-field and multifocal electroretinograms. *Clin Exp Optom*. 2017; 100: 668–675. 28266057 10.1111/cxo.12529

[103] Wan W, Chen Z, Lei B Increase in electroretinogram rod-driven peak frequency of oscillatory potentials and dark-adapted responses in a cohort of myopia patients. *Doc Ophthalmol*. 2019Oct 28. doi: 10.1007/s10633-019-09732-4 . [Epub ahead of print]. 31659575

[104] Liou SW, Chiu CJ Myopia and contrast sensitivity function. *Curr Eye Res*. 2001; 22: 81–84. 11402383 10.1076/ceyr.22.2.81.5530

[105] Stoimenova BD The effect of myopia on contrast thresholds. *Invest Ophthalmol Vis Sci*. 2007; 48: 2371–2374. 17460304 10.1167/iovs.05-1377

[106] Collins JW, Carney LG Visual performance in high myopia. *Curr Eye Res*. 1990; 9: 217–223. 2347202 10.3109/02713689009044516

[107] Williams KM, Verhoeven VJM, Cumberland P, et al. Prevalence of refractive error in Europe: the European Eye Epidemiology (E3) Consortium. *Eur J Epidemiol*. 2015; 30: 305–315. 25784363 10.1007/s10654-015-0010-0 PMC4385146

[108] Boscia F, Grattagliano I, Vendemiale G, Micelli-Ferrari T, Altomare E Protein oxidation and lens opacity in humans. *Invest Ophthalmol Vis Sci*. 2000; 41: 2461–2465. 10937554

[109] Palmquist BM, Philipson B, Barr PO Nuclear cataract and myopia during hyperbaric oxygen therapy. *Br J Ophthalmol*. 1984; 68: 113–117. 6691953 10.1136/bjo.68.2.113 PMC1040267

[110] Younan C, Mitchell P, Cumming RG, Rochtchina E, Wang JJ Myopia and incident cataract and cataract surgery: the Blue Mountains Eye Study. *Invest Ophthalmol Vis Sci*. 2002; 43: 3625–3632. 12454028

[111] Micelli-Ferrari T, Vendemiale G, Grattagliano I, et al. Role of lipid peroxidation in the pathogenesis of myopic and senile cataract. *Br J Ophthalmol*. 1996; 80: 840–843. 8942384 10.1136/bjo.80.9.840 PMC505624

[112] Simonelli F, Nesti A, Pensa M, et al. Lipid peroxidation and human cataractogenesis in diabetes and severe myopia. *Exp Eye Res*. 1989; 49: 181–187. 2767166 10.1016/0014-4835(89)90088-2

[113] Klaver CC, Wolfs RC, Vingerling JR, Hofman A, de Jong PT. Age-specific prevalence and causes of blindness and visual impairment in an older population: the Rotterdam Study. *Arch Ophthalmol*. 1998; 116: 653–658. 9596502 10.1001/archopht.116.5.653

[114] Eleftheriadis H, Amoros S, Bilbao R, Teijeiro MA Spontaneous dislocation of a phakic refractive lens into the vitreous cavity. *J Cataract Refract Surg*. 2004; 30: 2013–2016. 15342072 10.1016/j.jcrs.2004.04.060

[115] Ohno-Matsui K, Lai TY, Lai CC, Cheung CM Updates of pathologic myopia. *Prog Retin Eye Res*. 2016; 52: 156–187. 26769165 10.1016/j.preteyeres.2015.12.001

[116] Saunders LJ, Medeiros FA, Weinreb RN, Zangwill LM What rates of glaucoma progression are clinically significant? *Expert Rev Ophthalmol*. 2016; 11: 227–234. 29657575 10.1080/17469899.2016.1180246 PMC5898440

[117] Hattenhauer MG, Johnson DH, Ing HH, et al. The probability of blindness from open-angle glaucoma. *Ophthalmology*. 1998; 105: 2099–2104. 9818612 10.1016/S0161-6420(98)91133-2

[118] Chen SN, Ie B Lian, Wei YJ Epidemiology and clinical characteristics of rhegmatogenous retinal detachment in Taiwan. *Br J Ophthalmol*. 2016; 100: 1216–1220. 26659711 10.1136/bjophthalmol-2015-307481

[119] Haga A, Kawaji T, Tsutsumi T, Ideta R, Tanihara H The incidence of rhegmatogenous retinal detachment in kumamoto, Japan between 2009 and 2011. *J Clin Exp Ophthalmol*. 2017; 8: 2.

[120] Ivanisevic M, Bojic L, Eterovic D Epidemiological study of nontraumatic phakic rhegmatogenous retinal detachment. *Ophthalmic Res*. 2000; 32: 237–239. 10971186 10.1159/000055619

[121] Laatikainen L, Tolppanen EM, Harju H Epidemiology of rhegmatogenous retinal detachment in a Finnish population. *Acta ophthalmologica*. 1985; 63: 59–64. 3993347 10.1111/j.1755-3768.1985.tb05216.x

[122] Li X, Beijing G Rhegmatogenous Retinal Detachment Study Incidence and epidemiological characteristics of rhegmatogenous retinal detachment in Beijing, China. *Ophthalmology*. 2003; 110: 2413–2417. 14644727 10.1016/s0161-6420(03)00867-4

[123] Mitry D, Chalmers J, Anderson K, Williams L, Fleck BW, Wright A, Campbell H Temporal trends in retinal detachment incidence in Scotland between 1987 and 2006. *British journal of ophthalmology*. 2011; 95: 365–369. 20610474 10.1136/bjo.2009.172296

[124] Mitry D, Charteris DG, Yorston D, Siddiqui MAR, Campbell H, Murphy A-L, Fleck BW, Wright AF, Singh J The Epidemiology and Socioeconomic Associations of Retinal Detachment in Scotland: A Two-Year Prospective Population-Based Study. *Investigative Ophthalmology & Visual Science*. 2010; 51(10): 4963–4968. 20554615 10.1167/iovs.10-5400

[125] Polkinghorne PJ, Craig JP Northern New Zealand Rhegmatogenous Retinal Detachment Study: epidemiology and risk factors. *Clin Exp Ophthalmol*. 2004; 32: 159–163. 15068432 10.1111/j.1442-9071.2004.00003.x

[126] Törnquist R, Stenkula S, Tornquist P Retinal detachment. A study of a population-based patient material in Sweden 1971-1981. I. Epidemiology. *Acta Ophthalmol (Copenh)*. 1987; 65: 213–222. 3604613 10.1111/j.1755-3768.1987.tb07003.x

