# Supplementary material for: The Complications of Myopia: A Review and Meta-Analysis
Source: Invest Ophthalmol Vis Sci. 2020 Apr 29;61(4):49. doi: 10.1167/iovs.61.4.49 (PMC7401976; doi:10.1167/iovs.61.4.49)
Supplement: Supplement 1 [file iovs-61-4-49_s001.pdf]

# Supplemental material

**Table S1: Search terms used in PubMed**

| Complication | Search terms PubMed (from 1900-01-01 to 2019-06-01; species: humans; language: English) |
|---|---|
| **Myopic Macular Degeneration** | ("Myopia"[Mesh] OR "Myopia, Degenerative"[Mesh])<br>AND ("Cohort Studies"[Mesh] OR “Prevalence”[Mesh] OR “Incidence”[Mesh] OR “Case-Control Studies”[Mesh] OR “Cross-Sectional Studies”[Mesh])<br>AND ("Aged"[Mesh] OR "Middle Aged"[Mesh] OR “Adult”[Mesh])<br>AND ("Retinal Diseases"[Mesh]) |
| **Retinal Detachment** | ("Myopia"[Mesh] OR "Myopia, Degenerative"[Mesh])<br>AND ("Cohort Studies"[Mesh] OR “Prevalence”[Mesh] OR “Incidence”[Mesh] OR “Case-Control Studies”[Mesh] OR “Cross-Sectional Studies”[Mesh])<br>AND ("Aged"[Mesh] OR "Middle Aged"[Mesh] OR “Adult”[Mesh])<br>AND ("Retinal Detachment"[Mesh]) |
| **Cataract** | ("Myopia"[Mesh] OR "Myopia, Degenerative"[Mesh])<br>AND ("Cohort Studies"[Mesh] OR “Prevalence”[Mesh] OR “Incidence”[Mesh] OR “Case-Control Studies”[Mesh] OR “Cross-Sectional Studies”[Mesh])<br>AND ("Aged"[Mesh] OR "Middle Aged"[Mesh] OR “Adult”[Mesh])<br>AND ("Cataract"[Mesh]) |
| **Open Angle Glaucoma** | ("Myopia"[Mesh] OR "Myopia, Degenerative"[Mesh])<br>AND ("Cohort Studies"[Mesh] OR “Prevalence”[Mesh] OR “Incidence”[Mesh] OR “Case-Control Studies”[Mesh] OR “Cross-Sectional Studies”[Mesh])<br>AND ("Aged"[Mesh] OR "Middle Aged"[Mesh] OR “Adult”[Mesh])<br>AND "Glaucoma"[Mesh] |

**Table S2: Myopic Macular Degeneration classifications and definitions**

| Classification | Definition | Author (year) | Imaging |
|---|---|---|---|
| **a** | At least 1 of the following features: staphyloma, lacquer cracks, Fuchs' spot, or chorioretinal atrophy | Vongphanit et al. (2002) | Fundus photographs |
| **b** | M0, normal appearing posterior pole; M1, tessellation and choroidal pallor pattern; M2, posterior staphyloma; M3, lacquer cracks; M4, choroidal atrophy; M5, geographic atrophy and CNV | Avila et al. (1984) | Fundus photographs |
| **c** | 1: tessellated fundus, 2; diffuse chorioretinal atrophy, 3: patchy chorioretinal atrophy, 4: macular atrophy, and 'plus' lesions: lacquer cracks, myopic CNV and/or Fuchs spot. MMD was defined as ≥2. | Ohno-Matsui et al. (2015) | Fundus photographs |
| **d** | At least 1 of the following features: diffuse chorioretinal atrophy at the posterior pole, patchy chorioretinal atrophy, lacquer cracks, or macular atrophy | Hayashi et al. (2010) | Fundus photographs |

**Table S3: Prevalence of Myopic Macular Degeneration in high myopia studies**

| Author | Country | Ethnicity | Age, years* | SER (D)* | MMD | MMD definition (Table S1) |
|---|---|---|---|---|---|---|
| **Chen et al. (2012)** | China | Chinese | 40.6±17.1 (8-88) | -11.4±4.8 | 64.0% | c (≥2 excluding tessellation) |
| **Lai et al. (2008)** | Hong Kong | Chinese | 36.0±12.2 (>18) | -10.2±4.0 | 11.3% | a (excluding tessellation) |
| **Chang et al. (2013)** | Singapore | Chinese, Malay and Indian | - (>39) | - | 90.0% | b (≥M1; including tessellation) |
| **Koh et al. (2016)** | Singapore | Chinese | 21.1±1.2 (19-25) | -8.9±2.1 | 8.3% | c (≥2 excluding tessellation) |
| **Xiao et al. (2018)** | China | Chinese | 18.5 (7-70) | - 8.9 (-11.50, -7.63) † | 43.0% | c (≥2 excluding tessellation) |
| **Zhao et al. (2018)** | China | Chinese | 47.5±14.6 (>18) | -14.4±5.2 | 54.5% | c (≥2 excluding tessellation) |

* Mean±standard deviation (range); SER = spherical equivalent of refraction; D = dioptre; MMD = myopic macular degeneration.

† $25^{th}$ percentile, $75^{th}$ percentile

**Table S4:** **Best corrected visual acuity (LogMAR) in eyes with and without myopic macular degeneration**

| Author | Country | Age, years (mean) | BCVA with MMD (mean) | BCVA without MMD (mean) | P-value |
|---|---|---|---|---|---|
| Vongphanit et al. (2002) | Australia | 48.5 | 0.3 | 0.1 | <0.001 |
| Liu et al. (2010) | China | 56.9 | 0.25 | 0.06 | <0.001 |
| Gao et al. (2011) | Rural China | 51.9 | 0.3 | 0.1 | <0.001 |
| Chen et al. 2012) | Taiwan | 72.2 | 0.72 | 0.27 | 0.001 |
| Jonas et al. (2017) | Rural India | 49.0 | 1.38 | 0.11 | <0.001 |
| Shih et al. (2006) | Taiwan | 56.1 | 0.94 | 0.33 | 0.007 |
| Lichtwitz et al. (2016) | France | 60.0 | 0.74 | 0.25 | <0.001 |
| Zhao et al. (2018) | China | 47.5 | 0.62 | 0.17 | <0.001 |

BCVA = best corrected visual acuity, measured in logMAR

**Table S5 Characteristics of the studies investigating the relationship between retinal detachment and cataract extraction**

| Study | Study type | Follow up time (months) | Type of CE | Total participants (number of myopes) | RD in myopes (%) | RD in emmetropes (%) | OR (95%CI) | Age, years* | Refractive error / axial length (D/mm) * |
|---|---|---|---|---|---|---|---|---|---|
| Jeon et al. 2011 | Case control study | 7.27±±2.16 | phaco | 694†(347) | 6 (1.7) | 1 (0.28) | 6.1 (0.7-50.8) | 53.5±11.8 | 28.69±1.94 (case) 23.06±±2.17 (control) |
| Tsai et al. 2008 | Case series | 5.1-10.8 | ECCE with IOL, phaco met IOL | 52 (52) | 2 (3.84) | - | - | 61.3±13.2 | 28.22±1.64 |
| Ku et al. 2002 | Case series | 6 - 82 | ECCE with IOL, phaco met IOL | 125 (125) | 2 (1.60) | - | - | 61.58±12.27 | 28.45±±3.41 (ECCE) 28.45±±3.03 (phaco) |
| Fan et al. 1999 | Case series | 12 -89 | ECCE with IOL, phaco met IOL | 118 (118) | 2 (1.69) | - | - | 59.8±13.8 | 30.13±±2.08 |
| Alldredge et al. 1998 | Retrospective case series | 9- 77 | phaco | 80 (80) | 0 | - | - | 61.0 (33-85) | -10.0D ( -7.0D to -18.4D) |

| | | | | | | | | | |
|---|---|---|---|---|---|---|---|---|---|
| Gross et al. 1987 | Retrospective case series | ±±3-36 | ECCE with IOL | 117 (117) | 1 (0.85) | - | - | 69.2 | -10.0D±1.08 |
| Ripandelli et al. 2003 | Retrospective, paired-eye, case–control trial. | 36 months | ECCE | 1860 ‡ (930) | 74 (7.96) | 11 (1.18) | 7.2 (3.8-13.7*) | 62.5±8.5 | -20.7D±4.2 (ECCE)<br>-21.0D±3.8 (control) |

* Mean±standard deviation (range); †emmetropes as control group; ‡myopes without cataract extraction as controls; CE = cataract extraction; ECCE = extracapsular cataract extraction; RD = retinal detachment; D = diopter; mm = millimeters

**Table S6 Characteristics of the studies investigating the relationship between myopia and open angle glaucoma progression**

| Authors | Study type | Country | Total number of participants (number of myopes) | Male Gender (%) | Age (years)* | SER myopes (D)* | SER non-myopes (D)* | Follow-up, years* | Measurement modality of VF |
|---|---|---|---|---|---|---|---|---|---|
| **Ohno-Matsui et al. (2011)** | Retrospective, observational series | Japanese | 492 (492) | 41.5 | 40.6±16.6 | -13.4 (4.1) | - | 10.2±3.4 | Goldmann kinetic perimetry |
| **Perdicchi et al. (2007)** | Retrospective study | Italian | 294 (264) | 47.3 | 56.7±12.8 | NA (+1.75 to >-3D) | +8 to +2 | - (2-5.3) | Octopus 30° central field |
| **Lee et al. (2008)** | Retrospective study | Taiwan | 176 (176) 76 | 48.1 | 48.6±14.2 | NA ( -3D to <-9D) | - | 8.7±2.2 | Humphrey perimeter, 30-2 SITA standard program |
| **Doshi et al. (2007)** | Retrospective case series | Chinese | 14 (14) | 100 | 38.9 (25-66) | ≤-6 | - | 9.8±2.7 | Static automated white on white threshold perimetry (SITA standard) |
| **Chul Han et al. (2016)** | Retrospective comparative longitudinal cohort study | Korean | 232 (150) | | 45.7±11.8 | -4.5 (2.7) | -1.2 (2.3) | 9.9±2.6 | Humphrey Field Analyzer |
| **Yoshino et al. (2016)** | Retrospective matched case control study | Japanese | 140 (70) | 56.6 | 48.8±10.2 | -9.77 (2.50) | -1.62 (2.37) | 9.48±4.18 | Humphrey Field Analyzer |
| **Park et al. (2016)** | prospective observational study | Korean | 179 (101) | 45.8 | 70.2±15.8 | -3.8 (3.46) | 0.1±1.26 | 6.4±1.0 | Humphrey VF examination |

| | | | | | | | | | |
|---|---|---|---|---|---|---|---|---|---|
| **Lee et al. (2015)** | Retrospective cohort study | Korean | 369 (191) 151 | 52.0 | 61.4±12.1 | -1.77 (1.45) to -9.21 (3.57) | 0.71±1.04 | 4.4 | Humphrey Field Analyzer |

*mean±standard deviation (range); SER = spherical equivalent of refraction; VF = Visual Field; D = Diopters
