# Supplementary material for: The Complications of Myopia: A Review and Meta-Analysis
Source: Invest Ophthalmol Vis Sci. 2020 Apr 29;61(4):49. doi: 10.1167/iovs.61.4.49 (PMC7401976; doi:10.1167/iovs.61.4.49)
Supplement: Supplement 2 [file iovs-61-4-49_s002.pdf]

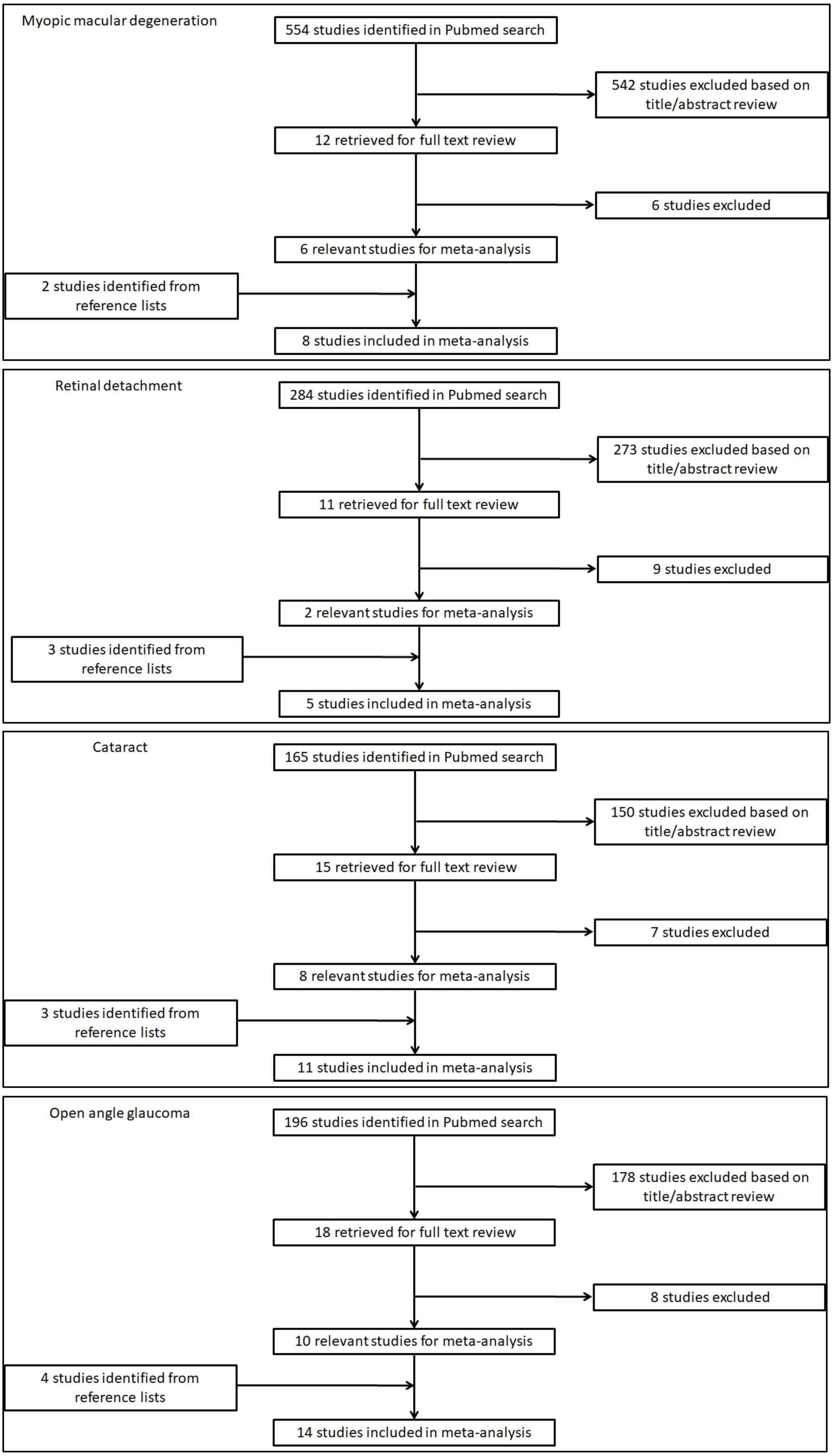
Myopic macular degeneration
554 studies identified in Pubmed search
542 studies excluded based on title/abstract review
12 retrieved for full text review
6 studies excluded
6 relevant studies for meta-analysis
2 studies identified from reference lists
8 studies included in meta-analysis
Retinal detachment
284 studies identified in Pubmed search
273 studies excluded based on title/abstract review
11 retrieved for full text review
9 studies excluded
2 relevant studies for meta-analysis
3 studies identified from reference lists
5 studies included in meta-analysis
Cataract
165 studies identified in Pubmed search
150 studies excluded based on title/abstract review
15 retrieved for full text review
7 studies excluded
8 relevant studies for meta-analysis
3 studies identified from reference lists
11 studies included in meta-analysis
Open angle glaucoma
196 studies identified in Pubmed search
178 studies excluded based on title/abstract review
18 retrieved for full text review
8 studies excluded
10 relevant studies for meta-analysis
4 studies identified from reference lists
14 studies included in meta-analysis

Supplemental Figure 1. Flow diagram of literature search and selection process of articles for meta-analyses of the association between myopia and myopic macular degeneration, retinal detachment, cataract and open angle glaucoma.
